# Indirect social influence and diffusion of innovations: An experimental approach

**DOI:** 10.1093/pnasnexus/pgae409

**Published:** 2024-10-01

**Authors:** Manuel Miranda, María Pereda, Angel Sánchez, Ernesto Estrada

**Affiliations:** Instituto de Física Interdisciplinar y Sistemas Complejos IFISC (UIB-CSIC), Palma de Mallorca 07122, Illes Balears, Spain; Grupo de Investigación Ingeniería de Organización y Logística (IOL), Departamento Ingeniería de Organización, Administración de empresas y Estadística, Escuela Técnica Superior de Ingenieros Industriales, Universidad Politécnica de Madrid, Madrid 28006, Spain; Grupo Interdisciplinar de Sistemas Complejos (GISC), Departamento de Matemáticas, Universidad Carlos III de Madrid, Leganés 28911, Spain; Grupo Interdisciplinar de Sistemas Complejos (GISC), Departamento de Matemáticas, Universidad Carlos III de Madrid, Leganés 28911, Spain; Instituto de Biocomputación y Física de Sistemas Complejos, Universidad de Zaragoza, Zaragoza 50018, Spain; Instituto de Física Interdisciplinar y Sistemas Complejos IFISC (UIB-CSIC), Palma de Mallorca 07122, Illes Balears, Spain

**Keywords:** social norms, social networks, diffusion of innovations, path-Laplacians, indirect social influence

## Abstract

A fundamental feature for understanding the diffusion of innovations through a social group is the manner in which we are influenced by our own social interactions. It is usually assumed that only direct interactions, those that form our social network, determine the dynamics of adopting innovations. Here, we test this assumption by experimentally and theoretically studying the role of direct and indirect influences in the adoption of innovations. We perform experiments specifically designed to capture the influence that an individual receives from their direct social ties as well as from those socially close to them, as a function of the separation they have in their social network. The results of 21 experimental sessions with more than 590 participants show that the rate of adoption of an innovation is significantly influenced not only by our nearest neighbors but also by the second and third levels of influences an adopter has. Using a mathematical model that accounts for both direct and indirect interactions in a network, we fit the experimental results and determine the way in which influences decay with social distance. The results indicate that the strength of peer pressure on an adopter coming from its second and third circles of influence is approximately two-third and one-third, respectively, relative to their closest neighbors. Our results strongly suggest that the adoption of an innovation is a complex process in which an individual feels significant pressure not only from their direct ties but also by those socially close to them.

**Significance Statement**

Adopting innovations is the engine of human civilization. We are continuously adopting ideas, behaviors, and technologies, which society perceives as new. In adopting an innovation, we are certainly influenced by the direct ties we have in our social network. However, as humans we also have a large capacity of imitating those who we perceive as socially close to us. Our research, designed to investigate theoretically and experimentally the role of these two types of social interactions—direct and indirect—shows that both of them must be taken into account. Indeed, adopting an innovation turns out to be the result of a complex combination of direct plus indirect social influences, involving differently our second and third circles of influence.

In a world of traditions, an innovation is an idea, practice, or object that is perceived as new by an individual (1, p. 12). The adoption of an innovation is not a trivial process, sometimes requiring a lengthy period of time. Once an innovation of interest arises, individuals and organizations often need to accelerate their adoption (2–5) for reasons that go from public health policies (6, 7) to marketing ones (8, 9). Therefore, understanding the diffusion of innovations, i.e. how innovations are “communicated through certain channels over time among the members of a social group” (1, p. 5) is of vital importance in many areas of social sciences research (8, 10–14). Starting with the seminal book of Rogers, first published in the 1960’s (1), several works have attempted to find mechanisms for accelerating the diffusion of innovations, either from the theoretical or the experimental point of view (3, 15–18).

A specific, but quite generic case of diffusion of an innovation relies on communication channels formed by interpersonal relations, by means of which an individual persuades others to accept a new idea (19–21). In principle, this kind of communication channel may be understood as a social network in which pairs of individuals are connected if they share an interpersonal communication (3, 22), be it face-to-face exchange or via e-communication, such as email or social media (23). However, as far as diffusion of innovations is concerned, the communication structure may be so complex that it goes beyond the interpersonal channels of communication recorded in the social network structure (24, 25). Indeed, as Rogers put it, “even the members of the system may not understand the communication structure of which they are part.” (1, p. 337). One of the reasons for which the network of interpersonal ties does not capture the totality of the communication channels is that individuals can learn from observation of other people’s behavior by means of nonverbal exchange of information. This mimicry of other’s behaviors conforms a phenomenon known as “social” or “observational” learning (26–28). In fact, even knowledge about certain statistics can act as a trigger of observational learning. One example is provided by Åberg (29) who cited the case of local demographic as an important influence on the risk of getting divorced. That is, knowing that some people not different from me have a certain behavior makes me copy them (30–33). Several similar examples are given in (34). Here, we consider that both verbal and nonverbal communication is transmitted through the edges of the network, which we will call direct communication. However, nonverbal communication can also occurs in an indirect way. That is, while the direct transmission between A and C in the path A–B–C implies the communication from A to B and then from B to C, the indirect communication can occurs from A to C without involving B.

A fundamental research problem is then how to account for the combination of interpersonal channels and observational learning into a unified network representation of communication channels for the diffusion of innovations. Here we take advantage of the seminal ideas of Granovetter (35) who assumes that all decision makers are influenced by everyone else in an “all see all” network. However, not everyone is equally considered in these interactions (36) as people often respond most to the behaviors of those similar to them in terms of common beliefs, education, socioeconomic status, and so forth (37). Thus, Granovetter (38) proposed that there is a range of ties with different strengths, where “the strength of a tie is a (probably linear) combination of the amount of time, emotional intensity, the intimacy (mutual confiding) and the reciprocal services that characterize the tie.” Here, we interpret these ideas as following. If *G* is a network such as (i,j) is an edge but (i,k) is not, then we consider the weighted complete network where (i,j) has weight equal to one and (i,k) has weight w<1, such that if w=0 we recover the original topology of the network (see Supplementary material). The problem is again how to quantify these strengths of ties. In this work, we use the ideas of Simmel about social distance (39). According to Simmel, social distance measures the nearness or intimacy that an individual or group feels towards another individual. Therefore, it is direct to associate the concept of social distance to that of communication proximity, due to their natural equivalence.

In this work, we analyze the diffusion of innovations by considering a situation in which an agent, who is influenced by their interpersonal ties in a social network, is also influenced by any other individual in the network with a strength that decays with the social distance separating them. Here, we consider the social distance to be exactly the number of ties that separate two individuals in their network of interpersonal channels, this is the shortest path distance between the two vertices representing them in the network. That is, the individuals connected to a given agent form their first circle of influences (see Fig. 1a). Those individuals separated by two ties form the second circle of influences, and so forth. Then, the traditional view of the process of diffusion in which an innovation is communicated through several iterations between agents directly interconnected by interpersonal ties, for instance, from A to B and then from B to C and from C to D (see Fig. 1b), is confronted with the model in which the diffusion process occurs via direct plus indirect influences, where the last take place via the second, third, etc. circles of influences (see Fig. 1c), such as A receives direct influence from B, but also indirect influences from C and D, where by direct we mean through the edges of the network and by indirect we mean “through-space” nonverbal interactions. The framework for our research is a study of the adoption of a drug between physicians in a hospital (40): based on this work, we designed and conducted a series of experiments to empirically measure how long-distance connections affect the adoption of innovations in a social network. Using the mathematical framework developed in Refs. (17, 41–43), we can measure the strength of such nondirect connections compared to the effect of direct friends in such adoption process. Therefore, we are following Rogers’ suggestion that “Alternative research approaches to post hoc data gathering about how an innovation has diffused should be explored.” (1). While some recent experiments have been done in this field (6, 13, 25, 44, 45), the effect of nondirect relationships has only been studied through data collection of studies not specifically designed to such purpose (24, 46). Therefore, our work fills the gap of experimental research explicitly designed to address the issue of nondirect connections on the diffusion of innovations.

**Fig. 1.**
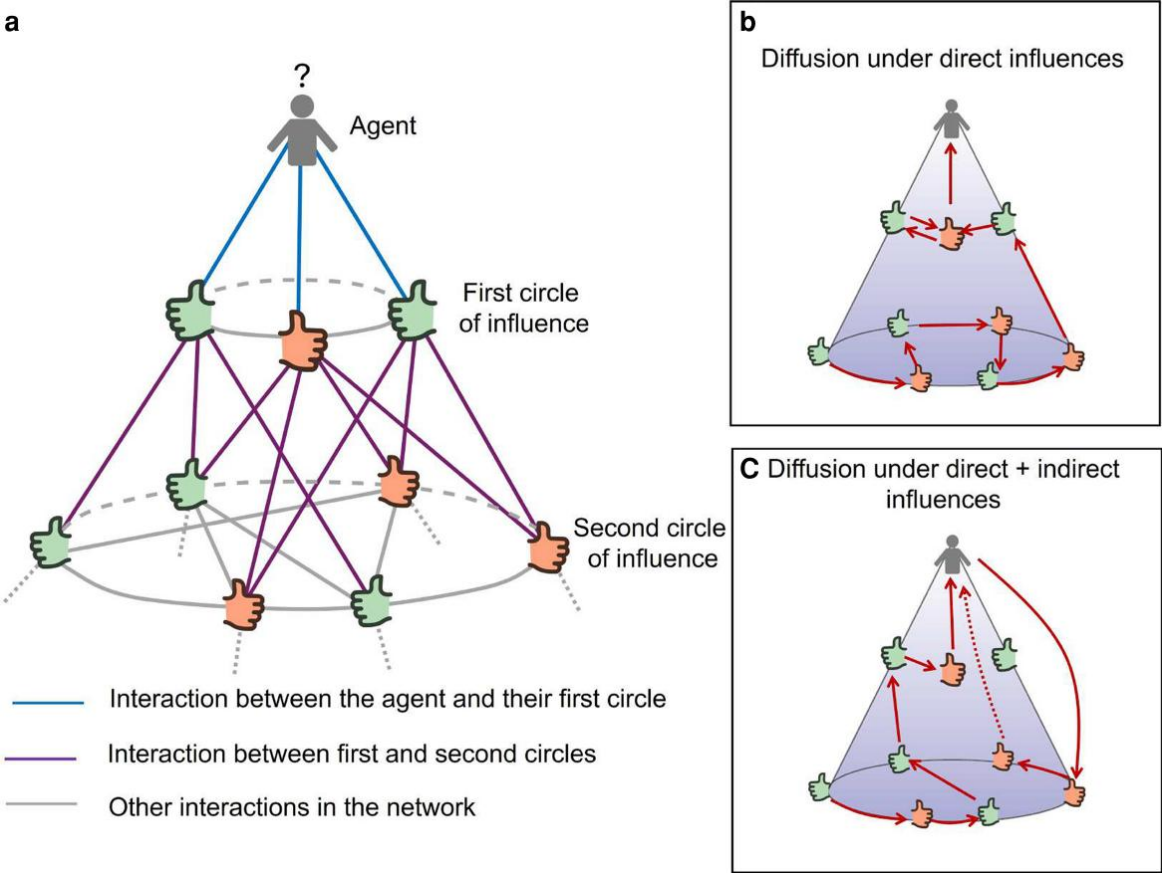
a) Representation of the cone of influences of an individual located at the top of the cone. The different kind of interactions are colored differently. b) Illustration of a hypothetical through-edges diffusive process occurring at the network. c) Illustration of a hypothetical diffusive process involving both through-edges and through-space interactions in the network.

## Experiment and model

### Experimental setup

As stated above, to investigate the existence and strength of influences—direct influences only or direct plus indirect ones—on the adoption of an innovation, we designed the following experiment. A group of participants is placed in all nodes of a network. At each round of the experiment, every participant has to choose between two colors, one which is assigned to the majority of participants and another one assigned only to a small number of them (but the respective fractions are not known to the participants). The participants receive a monetary reward (see Experimental methods from Materials and methods section for details) if they reach a global consensus in one of the two colors. The incentive is inversely proportional to the number of rounds they need to reach this steady state. In this scenario, in the initial round, the color of the majority represents the “tradition,” while the color of the minority represents an “innovation.” To stimulate the adoption of the innovation, participants receive a greater incentive if a consensus is reached on the initial minority color. Although the proportion of vertices with each of these colors evolves with time, we will refer to “majority” and “minority” throughout the experiment. In each session of the experiment, we randomly assigned some pairs of participants to role as friends. Pairs of friends form the edges which are created by imitating one network previously studied for the diffusion of an innovation in the real world (40).

**Fig. 2.**
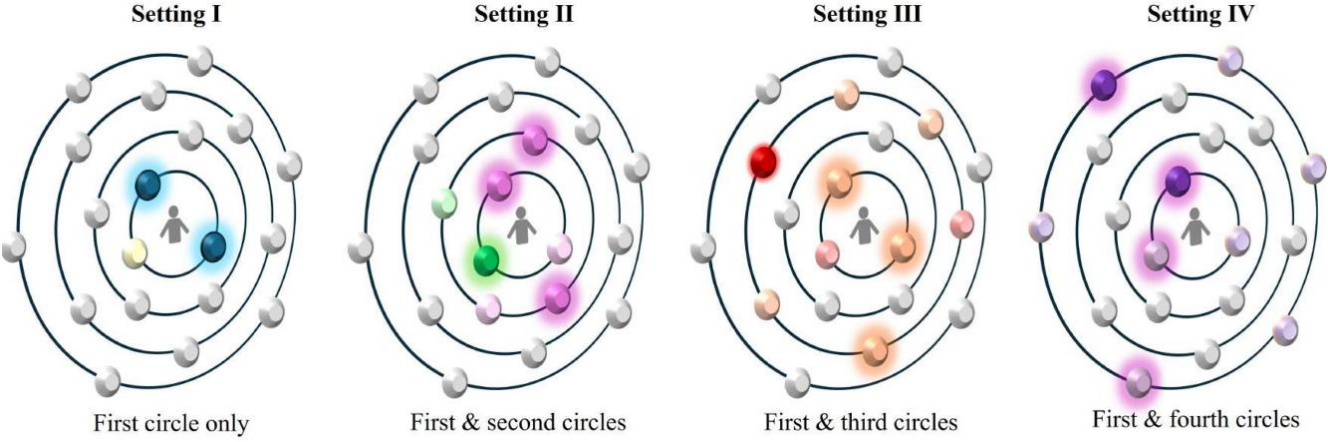
Schematic illustration of the experimental setup used in this work. The first setting consists in showing the participants information about the color chosen by two other subjects directly connected to them in the network. In the *k*th settings (k=2,3,4 for settings II, III, IV, respectively), the subjects observe the colors of two participants connected to them and two others in their *k*th influence circle. The colors in every setting change to avoid bias in the color selection: setting I (blue and yellow); setting II (magenta and green); setting III (orange and red); setting IV (purple and lilac). The first color in every pair is always the one of the “minority.” In the figure, the vertices observed by the subject are “illuminated” (with halo), while the others in the same circle are shown with pale colors (they are not observed at all by the subjects).

Let us now discuss how the initial choice of colors is assigned to the participants. Assuming that only 13% of the experimental subjects were early adopters, we initialized all sessions with 27 participants having the majority color and only 4 with the minority one. Once the participants have been assigned a color, the experiment takes place in four different settings. In each one of them, every subject sees a picture of the network, presented as an ego network centered at themselves, with vertices at longer distances being smaller than the ones which are nearer to them. The picture they observe is similar to the one in Fig. 2 where only limited information about the colors that every other vertex has is provided to them. The exact screenshots of the experiment can be found in the Supplementary material. A round finishes when all participants have made their choice. At the end of every round, participants see again a similar picture of a fixed amount of randomly chosen neighbors, with updated information about the colors they selected in the round.

In setting I, every subject had information about the colors of two of the agent’s nearest neighbors. In setting II, such information consisted of the colors of two of the agent’s nearest neighbors and two of the participants who are at distance two from the target. Similarly, for settings III and IV, the information provided was about two nearest neighbors and two in layer three or four, respectively (cf. Fig. 2). Every experimental session consists of a sequence of the four settings, each one comprising in turn of 13–15 rounds. A round is defined as the step in which every participant makes a decision, either keeping or changing the color currently assigned to that agent. Although participants knew that settings would end after at most 15 rounds, they did not know the exact maximum number of rounds used in each setting. In each session, the order of the settings was randomized to avoid order effects. Further details of the experimental design can be found under Experimental methods in the Materials and methods section and the Supplementary material.

### Theoretical model

In order to understand and analyze our experiments, we will compare them with the following analytical model (17, 41–43). Let us assume that every agent *i* has a propensity $u_{i}(0)=u_{i}^{0}$ to adopt an innovation at an initial time t=0. Then, the adoption of this innovation in a network is a consensus process in which the change of the state of subject *i* at a given time, $\dot{u_{i}}(t)$, is determined by


(1) $$\dot{u_{i}}\left(t\right)=\gamma_{\mathrm{NN}}\sum_{\mathrm{NN}}\left[u_{\;j}\left(t\right)-u_{i}\left(t\right)\right],$$

where $\gamma_{\mathrm{NN}}$ represents the “strength” of the nearest neighbors (NN) interactions, i.e. those vertices in the network directly connected among them. If we represent the states of every individual at a given time in the vector u(t), we can write


(2) $$\dot{u}\left(t\right)=-\gamma_{\mathrm{NN}}L_{\mathrm{NN}}u\left(t\right);\;u\left(0\right)=u^{0},$$

where $L_{\mathrm{NN}}$ is the Laplacian matrix of the network operating over the pairs of NN individuals. This is understood as an operator on a Hilbert space on the set of vertices of the network acting on a function *f* defined in the same space and evaluated on the vertex *v* as: $\left(L_{\mathrm{NN}}f)(v)\;:\;=\sum_{\mathrm{NN}}[f(w)-f(v)\right]$, where the sum is over all the NN of *v*.

The solution of this equation is: $u(t)=e^{-t\gamma_{\mathrm{NN}}L_{\mathrm{NN}}}u^{0}$, and the steady state is the one in which every vertex has a state equal to the average of the initial condition. This equation represents the diffusion of the adoption of the innovation across the network, assuming that the process is continuous in time as well as in which the state of the individuals may take a continuous range of values. However, in an experimental setup as the one described before the time is discrete as it is determined by the rounds taken to reach the consensus and the results are binary, i.e. an agent either adopt or does not adopt the innovation. Therefore, here we discretize time as follows. For any given time *T* and a number of rounds r, we equidistribute *r* points in the interval [0,T], such that the discretized solution is equal to the continuous solution at those times. We also proceed to discretize the output of the model by introducing a threshold parameter: $△\cdot u_{i}(t_{c})$, where △∈[0,1] and $u_{i}(t_{c})$ is the state of the vertex *i* when the consensus was reached, which is equal to the average of the entries of $u^{0}$. This means that when an agent has a propensity to adopt the innovation larger than this threshold it is assumed that the agent adopts the innovation. Otherwise, it is assumed that it has not adopted the innovation.

To account for the influence of the individuals in the second circle of influence of the agent *i*, we can define the Laplacian operator $\left(L_{\mathrm{NNN}}f)(i)\;:\;=\sum_{\mathrm{NNN}}[f(j)-f(i)\right]$, where now the sum is carried out over all next nearest neighbors (NNN) of *i*, i.e. those separated by two edges in the network. Similarly, we can extend this definition to the third, fourth and so for NN of a given agent, such that we can write the innovation diffusion model as:


(3) $$\begin{array}{rl} \dot{u}\left(t\right) & =-\gamma_{\mathrm{NN}}L_{\mathrm{NN}}u\left(t\right)-\gamma_{\mathrm{NNN}}L_{\mathrm{NNN}}u\left(t\right)-\cdots -\gamma_{D}L_{D}u\left(t\right);\\ u(0) & =u^{0}, \end{array}$$

where $\gamma_{\mathrm{NNN}}$ is the “strength” of the interactions between next nearest neighbors and *D* designates the diameter of the network, i.e. the longest shortest path between any pair of vertices. The intuition dictates that the strength of the interaction decays with the separation between the pairs of agents in the network, i.e. $\gamma_{\mathrm{NN}}>\gamma_{\mathrm{NNN}}>\cdots >\gamma_{D}$. In the experiments designed in this work the diameter of the network is five, i.e. D=5, and we can use the following notation accordingly: $c_{1}=\gamma_{\mathrm{NN}};c_{2}=\gamma_{\mathrm{NNN}};\ldots$. Similarly, we designate $L_{1}=L_{\mathrm{NN}}$; $L_{2}=L_{\mathrm{NNN}}$, etc., where, as defined before, $\left(L_{d}f)(v)\;:\;=\sum_{d(v.w)=d}[f(w)-f(v)\right]$. Let us fit $\gamma_{\mathrm{NN}}=c_{1}=1$, such that we can write:


(4) $$\dot{u}\left(t\right)=-\left(L_{1}+\sum_{d=2}^{D}c_{d}L_{d}\right)u\left(t\right);u\left(0\right)=u^{0}.$$

## Experimental results

We recruited 592 participants from the IBSEN subject pool at Universidad Carlos III de Madrid (UC3M) to participate in a series of 21 experimental sessions. All our experiments were approved by the ethical committee at the UC3M. Informed consent was obtained from all participants involved in our study, in strict compliance with the principles expressed in the Declaration of Helsinki.

The average age was 30.4 years (median 25, mode 22). The gender representation was 63.8% female, 35.9% male, and 0.3% nonbinary. The distribution of gender and age through the experimental sessions is shown in the Table S1 and Figs. S14 and S15.

To analyze the experimental results from a realistic perspective, we considered that a subject who has adopted the “minority” color becomes an adopter of the innovation from that round on. This definition is intended to take into account the differences in time between the experimental settings and the real adoption of an innovation. While the first takes minutes, the second can take years, and once a subject has adopted an innovation in the real world, it will take long time until they can abandon it, in case they ever do so. In 14 of the 21 experimental sessions, the participants reached consensus in setting I, and for settings II–IV, the global consensus was reached in 16 of the 21 sessions (see Fig. S9). This means that in some of the 21 experiments there was at least one individual (stubborn) who did not join the consensus of the group for the duration of the setting. In total, there were 11, 6, 10, and 8 stubborn individuals in settings I, II, III, and IV, respectively, which clearly points out to the lack of any bias in the number of such individuals in relation to the type of social interactions considered in the experiments.

**Fig. 3.**
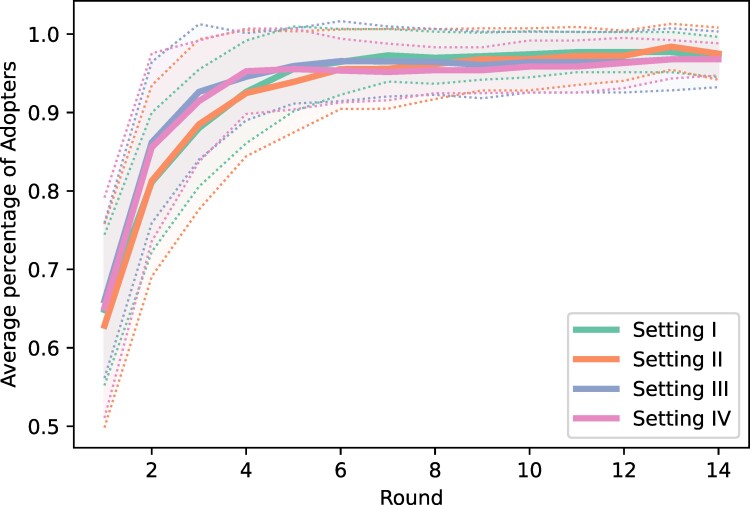
Average percentage of adopters (including bots) as a function of the experimental rounds, per setting. Shaded areas represent standard deviations.

In Fig. 3, we illustrate the cumulative distributions of the proportion of adopters of the innovation versus round for each of the four settings considered here and averaged over the 21 experimental sessions. In sessions where most participants reached consensus in round *x* but some were stubborn, we adjust the Fig. 3 plot to show global consensus at round *x* + 2 for aesthetic purposes. In general, the percentages of adopters in the second round are approximately the same for the four settings (for round I all the percentages are exactly the same as we initialize all the experiments with this percentage of adopters). However, for rounds 3–5 these percentages show the largest differences between the four settings. In round 3, the percentage of adopters in setting I is about 84.5%, while for settings II and III it grows to 88.8%, and for setting IV it is 85.4%. In round 4, these percentages are: 89.2, 92.9, 93.4, and 91.1%, respectively, and for round 5 they are: 92.9, 96.2, 96.2, and 94.2%. Although these values are average percentages that may be hiding the specifics of each session, (see further analysis), they clearly indicate an acceleration in the number of adopters in settings II–IV relative to setting I, particularly for settings II and III. We would like to remark that these results seem to point to the fact that the second and third neighbors of an agent significantly influence their decision in choosing an innovation. Such influence seems to drop for the fourth circle of influences.

Let us now discuss the fit of the experimental results to our model. To that end, we proceed by considering the individual experiments. For each setting, we fit the results of each of the experimental sessions to find the parameters $c_{2},$  $c_{3}$, and $c_{4}$ as well as to find the values of △ (the value of u(t) that triggers the adoption of the innovation) and *T* (the time equivalent to the number of rounds in the experiment) that best fit the data as detailed in Fitting method in Materials and methods section.

**Fig. 4.**
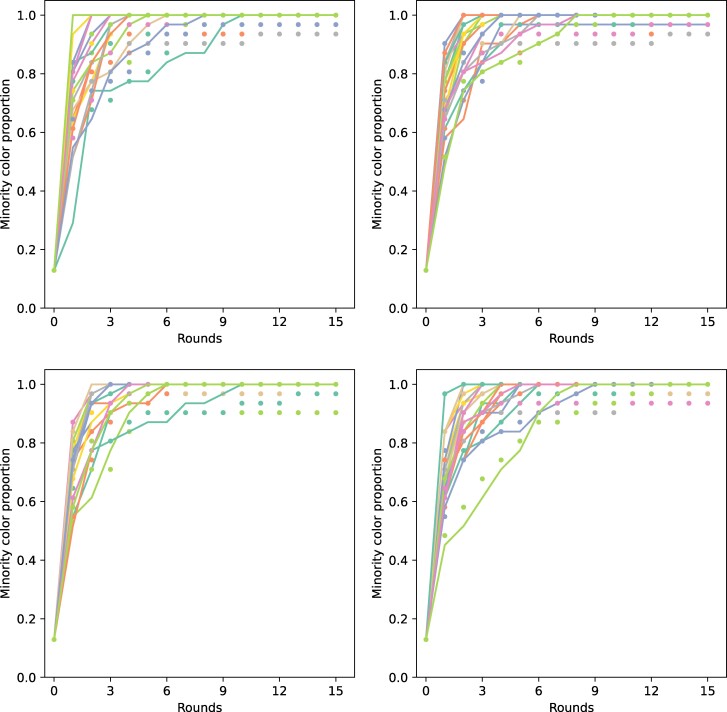
Proportion of adopters (dots) and their theoretically expected behavior (solid lines) as a function of the rounds for all the experimental sessions (in colors) and the different settings: setting I in upper left subplot, setting II in upper right subplot, setting III in lower left subplot and setting IV in lower right subplot.

In Fig. 4, we illustrate the results of the fitting procedure for the four settings in the 21 experiments. The experimental data are visualized as points of colors representing each experimental session. The best fits obtained by the procedure described previously are illustrated as curves of the same colors as those of the data points. As can be seen in the plots, the fittings are much better for the initial times of the time evolution of the adoption procedure than for the final ones. The reason is that, as mentioned before, in several experimental sessions, there were stubborn participants who never joined the consensus state followed by the large majority of subjects. On the contrary, the diffusion model assumes that every participant is predisposed to reach the consensus state. In any event, we have analyzed our experimental data by removing outliers that are basically coincident with the presence of stubborn subjects, and the results are approximately the same (see the Analysis of outliers and stubborn individuals in Materials and methods section). As there are theoretical models that take into account the presence of stubborn participants, we maintain the general idea of using a diffusion model as our main goal here is to investigate the role of indirect peers pressure in the adoption of innovations. Further studies can be designed to study models in which stubborn participants are explicitly considered.

**Fig. 5.**
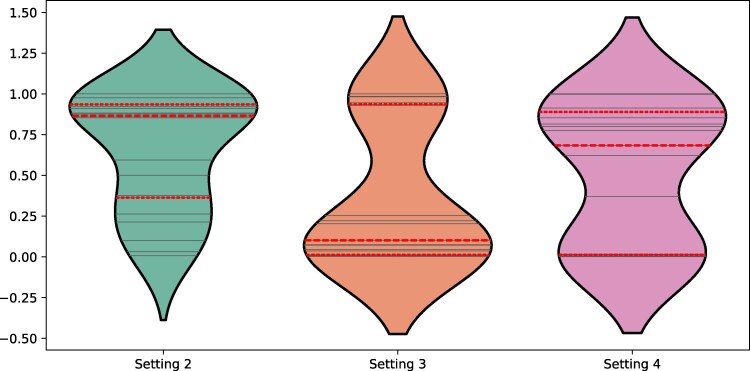
Violin plot of the distribution of fitted parameters for each of the Settings that include indirect peer-pressure. Solid lines are the individual value of the fitted parameter for each of the sessions, while the dashed lines are the interquartile ranges of each distribution.

From the perspective of accounting for the direct and indirect influences of peers on the adoption of an innovation, the parameters $c_{d}$ are the most relevant. In Fig. 5, we illustrate the distributions of the parameters $c_{2}$ (setting II), $c_{3}$ (setting III), and $c_{4}$ (setting IV), obtained from the best fittings of the experimental data to the models of direct plus indirect influences on the network. The values of the mean and standard deviations of these coefficients are as follows: $c_{2}=0.651\pm 0.354$; $c_{3}=0.373\pm 0.427$; $c_{4}=0.513\pm 0.420$. We recall that the strength of direct influences is $c_{1}=1$.

We then check whether the differences between the means of these coefficients are significant according to their *P*-values, i.e. the probability of obtaining the observed difference between the samples if the null hypotheses were true. The null hypothesis states that the difference between the averages is 0, that is, there is no difference. We obtained $p(c_{2},c_{3})=0.0269$, $p(c_{2},c_{4})=0.256$, $p(c_{3},c_{4})=0.290$. Therefore, the only significant difference, i.e. P<0.05, is between the coefficients that represent the influences of the second and third circles, but not between the second or third with the fourth, where there is no empirical evidence to reject the null hypothesis.

This lack of significant difference between the means of $c_{4}$ and the other two coefficients could be due to several experimental factors that cannot be explained with the information that we have obtained from them. Consequently, we eliminate the results concerning the influence of the fourth circle of influence and focus on the fact that our results indicate that there is a relatively large influence of the second NN on the adoption of an innovation by an agent, which is on average 65% as strong as the direct influence of peers, and a relatively small, but not negligible, influence of the third NN, which is on average 37% as strong as the direct interaction. We can then write an approximate model that describes the results of our experiments as follows:


(5) $$\begin{array}{rl} \dot{u}\;\left(t\right) & \approx -\left(L_{1}+\left(0.651\pm 0.354\right)L_{2}+\left(0.373\pm 0.427\right)L_{3}\right)u\;\left(t\right);\\ u(0) & =u^{0}. \end{array}$$

The empirical model 5 reflects the fact that the strength of the influences decays with the increase in the social distance (measured here as the shortest path) between the subjects. This model can be approximated very well by considering that the coefficients $c_{d}$ are indeed a linear function of the distance, such that we can write:


(6) $$\dot{u}\left(t\right)\approx -\sum_{d=1}^{D}\left[\left(\frac{4-d}{3}\right)L_{d}\right]u\left(t\right);\;u\left(0\right)=u^{0}.$$

Using this approximation, we can say that the strength of the interactions between a subject and its second circle of influences is about two-third of that with their closest neighbors, and those in the third circle have an influence which is about one-third of the ones between NN. Whether this is a general expression for other cases of diffusive adoption of innovations is something which should be taken with prudence and analyzed in individual cases. Linear decay models have previously been used to consider social effects, such as rumor transmission in a network, where an exponentially truncated linear decay function is used to characterize the decay such that if the acceptance time is small, the decay function is dominated by a linear decay function (47) (see also (48, 49)). Other kinds of decay are also studied in the Supplementary material.

To gather further evidence on the role of nondirect influences, we now focus on the individual decisions. We want to unveil which pieces of information are people using to make their decisions (whether to choose the innovation color or not). In order to do so, we study the problem as a classification problem, where our aim is to predict the color a person chose as function of the information available: whether people had the innovation as their initial color, whether the innovation color is the majority color seen, the percentage of their first neighbors with the innovation color, and the percentage of *n*-distance neighbors with the innovation color. Then, the chosen color is the dependent variable and the four pieces of information are the features or independent variables. We use random forests (RF) as classification technique and the feature importance analysis (see the Random forests and feature importance analysis in Materials and methods section for details).

**Fig. 6.**
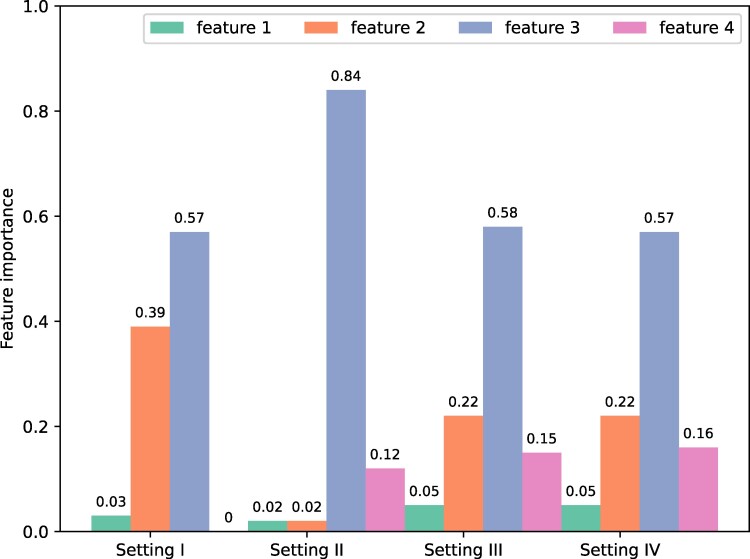
Feature importance analysis per experimental setting. The features are (1) innovation as initial color, (2) innovation as majority color seen, (3) percentage of first neighbors with the innovation color, and (4) percentage of n-distance neighbors with the innovation color.

Our first result is that the color that a person chooses in each round can be predicted with high accuracy (84%). Subsequently, when we study the feature importance of this classification problem, i.e. the contribution of each piece of information to predict people’s decisions, we see that the importance of the four pieces of information is different depending on the experimental setting (see Fig. 6).

As can be seen in the plot, the initial color assigned to the participants (feature 1) is irrelevant to people’s decisions. In the first setting, participants have no information of their neighbors at distances bigger than one, and hence feature is being unimportant in this setting. The most important feature is whether the direct neighbors shown to them have acquired the innovation or not. It is twice as important as the majority opinion (feature 2). In settings II to IV, the information of *n*-distance neighbors is relevant for the decisions, and, notably, this information becomes very important. In setting II, with direct neighbors and neighbors at distance two, the most important variable is still the direct neighbors information; four times more important than the 2-distance information. In this setting, the information of the majority (feature 3) is irrelevant. In settings III and IV, there is a decrease in the importance of the first neighbors information in favor of the importance of the majority and the *n*-distance neighbors information. This suggests that people are taking the whole picture into account when making their decisions.

It is worth noting that we cannot discard the potential effects of visual biases in the results observed here. It has been previously shown that potential biases can be introduced in experiments due to the different operational mechanisms for color and location, as well as for the increment in complexity due to combined use of shape and color (although here we maintain the same shape of the figures) (50). Other potential sources of biases include the observed fact (51) that objects that are highly salient and stand out from the background may immediately receive attention priority. Therefore, further experiments including potential psychological biases may be designed to investigate these potential factors.

In general, the analysis suggests that the *n*-distance information influences the adoption of innovations, and this is more relevant as the information present is from higher distances.

## Discussion

**Fig. 7.**
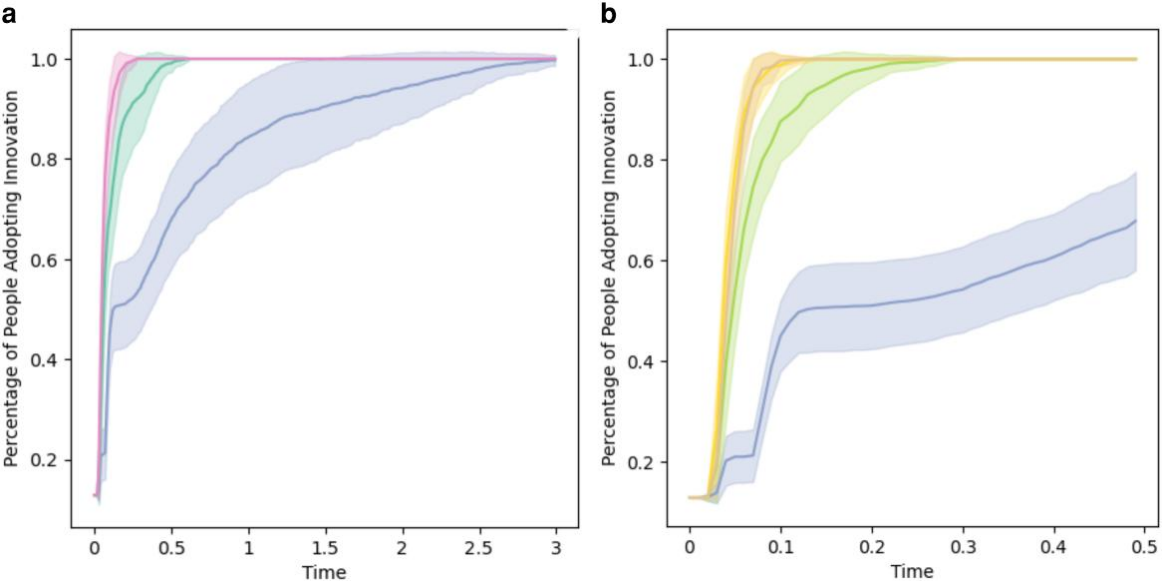
Comparison between different laws for indirect peer pressure, averaging over 1,000 initial conditions. In the figure in the left, the bottommost line represents the only direct pressure diffusion. The central line corresponds to considering also indirect pressure from next nearest neighbors, while the topmost line considers pressure from all neighbors at distance less or equal than 3. In the right figure, the bottom line is again the only direct diffusion. The middle line corresponds to the law $c_{d}=\frac{4-d}{3}$ commented in section Experimental results. The two topmost lines, almost identical, represent two laws that accelerate the spreading of the innovation, using the laws $c_{d}=\frac{5-d}{4}$ and $c_{d}=\frac{2}{1+d}$, respectively from bottom to top.

In this article, we have provided solid evidence pointing out to the fact that indirect influences play a fundamental role in the diffusion of innovations, a key process in a globalized technological society such as the current one. The results of an experiment specifically designed to probe into this question demonstrate that, as summarized in Fig. 7, the adoption of the innovation by about 60% of participants in our experiments may take around five times less steps if we allow them to see the influence of those socially close but not connected to them. The situation is even more dramatic if we consider the times at which 80% of the experimental subjects adopted the innovation. In this case, the reduction of time is more than 10-fold under the indirect influence of peers: In practical terms, this means that an innovation which would take around a year to be adopted under direct influences only, would be adopted in about 1 month under the joint effect of direct and indirect influences. We note also that the diffusion of innovation goes faster in the first stages of the diffusion process if information on long-range distance is present; see settings III and IV curves in Fig. 3. Further, independent evidence that information is indeed the mechanism behind the acceleration of diffusion of the innovation comes from a feature analysis that reveals the way participants weigh their knowledge of the social context. All in all, the experimental evidence sends a clear message with practical implications: diffusion of innovations can only be properly understood if the influence of people at different levels of social distance is taken into account.

As an additional illustration of how such indirect influences can change the rate of diffusion of an innovation, we do the following theoretical experiment. By considering the same network studied here experimentally, we tune the indirect influences without changing the direct interactions between the agents. We tune these influences simply by changing the coefficient $c_{d}$ which determines the weight that the not-direct influences have on a given agent. In Fig. 7, we illustrate the results where we also plot the experimental results obtained here for no indirect influences as well as for direct+indirect ones, in which the coefficient fitted to the experimental data is $c_{d}=\frac{4-d}{3}$. When we increase such indirect influences to $c_{d}=\frac{5-d}{4}$ or to $c_{d}=\frac{2}{1+d}$, the results are obvious: a significant increase of the diffusive dynamics in which the times to adopt the innovation are significantly reduced.

One interesting question arising from our experimental results is the lack of differences between the coefficients for the fourth neighbor influence and the coefficient for the second and third neighbors. While, as already mentioned, this may be an effect of sample size or, perhaps, of the network size, it may also be the case that the weight we give to the influence of socially distant contacts saturates, meaning that beyond the first two or three layers of contacts we take in the input of further ones in the same manner. This might arise as a consequence of limited cognitive capabilities: in a general situation in the population at large, we will have many more contacts as social distance increases, and we are thus led to consider them in a less specific manner. Remarkably, these results coincide with those reported by Christakis and coworkers who observed that the risks of spreading obesity (52), smoking behavior (53), happiness (54), and alcohol consumption behavior (55) are influenced by individuals up to three degrees of separation between each other. They observed that by the fourth degree of separation there was no excess relationship between individuals in the large social network analyzed over a period of 32 years. Further experiments in larger networks would help clarify this point, although it must be taken into account that would require a large sample of subjects that would play simultaneously with the logistic challenge that implies (56).

In terms of real-world impact, our results indicate that acting on indirect influences could change very significantly the adoption rates of innovations. Mass media has been frequently identified as a principal actor of indirect influences. By this means, for instance, teenagers in one country can observe the attitudes and behaviors of others in a different one, copying them for good or for bad. Therefore, mass media can act as a modulator of indirect influences, which may change significantly the dynamics of the adoption of innovations. While this is a source of influence which is not really amenable to use as an intervention, other approaches may lead to specific actions in order to increase, e.g. the adoption of socially desirable behaviors. Akin to behavioral interventions in which people are informed of the expectations of others on their behavior (57), we could think of campaigns in which subjects of interest receive information on what other, socially distant, people do in the relevant context. Our experimental results indicate that giving only a limited amount of information about second- or third-order contacts could already lead to highly increased rates of behavior adoption.

In closing, the results found in this work clearly point out to the fact that when adopting an innovation we are not only influenced by peers directly connected to us in our social networks, but that we are also significantly influenced by people socially close but not directly connected to us in any of our social networks. This work paves the way to the development of further experimental and theoretical setting which will allow us a better understanding of the dynamics underneath the diffusion of innovations.

## Materials and methods

### Experimental methods

The experiment consisted of four treatments, which we refer to as “settings” to highlight our interest in informational settings, to study the influence of peer pressure on consensus. Here we summarize a few additional details that are needed to complete the definition of the experimental setup. The colors in each setting were different to avoid learning biases. The eight colors were setting I (blue and yellow); setting II (magenta and green); setting III (orange and red); setting IV (purple and lilac). The first color of each pair being the minority. In case one or more subjects did not make a choice in a given round or were missing, we declared them “inactive.” Then, to avoid any change in the structure of the underlying network, we replace that player(s) with a “bot,” which is programmed to have 50% probability of choosing color at random and 50% of following the majority of the color they would see. Nonetheless, bots were clearly marked as inactive subjects and were not shown to active participants if possible. The original network from the study (40) was slightly modified so that every node had at least two nodes at distance one and two nodes at distance four. This was done by removing just one edge and adding another one (edge removed from node 10 to node 30 and added edge between nodes 2 and 17 from the Pajek dataset Galesburg drug study 2, friendship network (58)).

Participants received points that, at the end, were converted into money. Each participant received 1 point per active round (if they made a decision before the timeout occurred). Then, if consensus was achieved, all of them received 5 points per each round left until round 15 (maximum possible number of rounds) if they were active at least in one of the two last played rounds of that setting.

These experiments were programmed using the Python package oTree (59) version 5.10.3, using Cytoscape.js (60) version 3.1.0 for graph visualization. The code is available in (61). Data results are available in (62). Snapshots of the webpages presented to the participants are shown in the Supplementary material.

### Fitting method

For a given experiment and a specific setting, we obtain the vector $u_{exp}$ with the percentages of adopters in each round, as well as the initial condition vector $u_{0}$. Then, for each triplet of parameters $\left(c_{d},△,T\right)$, we produce a prediction vector $u_{\mathrm{pred}}$ based on our model using the following method. From the interval [0,1], discretized in intervals of size 0.01, we choose $c_{d}$ and calculate the solution of the dynamical system, obtaining the solution $u(t)=[\text{exp}(-t(L_{1}+c_{d}L_{d}))]u_{0}$. Now, we fix a value for *T* taken between 10 and 1,000 in steps of size 10, and we identify each round *i* of the experiment with time $t_{i}\in [0,T]$ such that these times are equally distributed throughout the interval. Finally, we use one of the threshold values △∈{0.4,0.5,0.6,0.7,0.8,0.9,0.99} and calculate each entry of the vector $u_{\mathrm{pred}}$ by summing all the entries of $u(t_{i})$ above the threshold ($u_{\mathrm{pred}}(i)=\mathrm{sum}(u(t_{i})>△)$. Hence, for each combination of $\left(c_{d},△,T\right)$, we obtained the prediction $u_{\mathrm{pred}}$, which we can compare to the real proportion of adopters during the experiments ($u_{\exp})$ by calculating the mean square error (MSE) and the Pearson correlation coefficient between the two vectors. The best fit is the combination of parameters that minimize MSE.

### RF and feature importance analysis

RF (63) are one of the most effective algorithms, excelling in predictive performance in various application domains, while demonstrating robustness against overfitting and internal correlations among explanatory variables. RF employ decision trees with a unique ensemble technique called bootstrap aggregation (bagging). Unlike traditional decision trees, RF combines the results of multiple weak learners using bagging, which aggregates results through averaging in regression tasks and a voting system in classification tasks. One notable feature of RF is its utilization of “Out-Of-Bag” (OOB) data, which comprises approximately one-third of the original dataset that is not used in constructing each tree. OOB data serve as a test data set to estimate misclassification error and can also be used to analyze the relative importance of each feature in the classification problem. For every tree in the forest, the *j*th feature of the OOB sample is randomly permuted, and the resulting increase in OOB error is computed. This increase serves as a measure of the importance of the *j*th feature for correct classification: the greater the increase in OOB error, the more critical the variable is for achieving accurate classification. This analysis provides valuable insights into the contribution of each variable to the classification process, helping in feature selection and interpretation. To estimate the accuracy of the classifier, we used nested cross-validation (NCV) (64). NCV operates by employing cross-validation (CV) within two sequential loops: an inner loop for hyperparameter selection and an outer loop for computing test error. In our experiments, both the inner and the outer loops utilized five-fold CV.

### Analysis of outliers and stubborn individuals

It is evident that, during the realization of experiments, several uncontrollable factors may produce outliers which deviate from the statistical behavior of the majority. In order to detect such individuals, we tested the results against three different methods for outlier detection: *Z*-score, Tukey method, and mean regression (MR). The first two methods are well documented in the literature (65, 66), so we need to explain the last method. MR consist in measuring how much the mean changes when we remove the current value. High scores corresponds to values that change notoriously the mean and can be considered an outlier.

In Table 1, we give the number of experiments that the corresponding method detected as an outlier. In parentheses, we give the number of stubborn individuals that were present in such experiments. Then, in setting II all outliers identified by Tukey method coincide with those in which there is at least one stubborn individual. In setting III, Tukey and MR identify six outlier sessions all of which have stubborn participants (six out of eight in this setting). Finally, the eight stubborn participants that appear in setting IV are present in the experiments identified by MR as the outlier sessions. Therefore, the statistics of the results clearly point out the identification of those experiments in which there are stubborn participants as outliers, which is what it should be expected by considering that the assumption of the diffusion model is the whole predisposition of agents to reach the consensus state.

**Table 1.** Table showing the outlier rounds detected by the different methods.

Setting	*Z*-score	Tukey	MR
II	5 (1); 9 (1)	2 (1); 5 (1); 9 (1); 16 (2); 20 (1)	2 (1); 5 (1); 9 (1); 16 (2); 20 (1)
III	7 (1)	1 (3); 2 (1); 5 (3); 7 (1)	1 (3); 2 (1); 5 (3); 7 (1)
IV	None	None	3 (1); 6 (1); 9 (0); 16 (2); 20 (3)

The numbers in parentheses represent the number of stubborn subjects for that session and setting.

Once we have identified the statistical outliers in the experiments, we proceed to recalculate the models after their removal. The new coefficients are $c_{2}=0.707\pm 0.319$; $c_{3}=0.431\pm 0.453$; $c_{4}=0.503\pm 0.411$. We also recalculate the *P*-values for the three pairs of coefficients and obtain: $P(c_{2},c_{3})=0.0275$, $P(c_{2},c_{4})=0.0798$, $P(c_{3},c_{4})=0.589$, which indicates that the means of $c_{3}$ and $c_{4}$ are less different than before and, although $P(c_{2},c_{4})$ is significantly smaller than without eliminating outliers, it still is not significant at 95% of confidence. The empirical model without the statistical outliers (excluding those detected by MR) is then:


(7) $$\begin{array}{rl} \dot{u}\left(t\right) & \approx -\left(L_{1}+\left(0.707\pm 0.319\right)L_{2}+\left(0.431\pm 0.453\right)L_{3}\right)\;u\;\left(t\right);\\ u(0)\;= & u^{0}. \end{array}$$

which increases slightly the strength of the influence of the second and third circle of influence in relation to the case where the outliers were not removed. All in all, the results indicate that the statistical outliers do not significantly affect the general findings that long-range influences play a fundamental role in the diffusion of innovations across a network of social interactions. Guided by the fact that most of the outliers are those having stubborn individuals, we conducted a final calibration of the model by removing all experiments in which there was at least one of these individuals. The results are very similar to those obtained when removing the outliers detected by MR and are not reproduced here.

## Supplementary Material

pgae409_Supplementary_Data

## Data Availability

The data obtained from the experiment sessions are available at (62). The codes used for obtaining the results presented in this article are available at (61).

## Preprints

This manuscript was posted on a preprint:arxiv.org/abs/2407.01106
