# Supplementary material for: Indirect social influence and diffusion of innovations: An experimental approach
Source: PNAS Nexus. 2024 Oct 1;3(10):pgae409. doi: 10.1093/pnasnexus/pgae409 (PMC11452798; doi:10.1093/pnasnexus/pgae409)
Supplement: pgae409_Supplementary_Data [file pgae409_supplementary_data.pdf]

Supporting Information Text

1. The nature of the “all-see-all” representation

To illustrate the significance of the representation adopted in the paper of the concept of “all-see-all” stated by Granovetter we will consider here an example. Let us consider 6 skaters which form a social network reflecting their mutual influence in their skating styles. The ties in this network can represent friendship, advice or any other type of mutual influence. Let us assume that the network has the structure illustrated in Fig. S1(a).

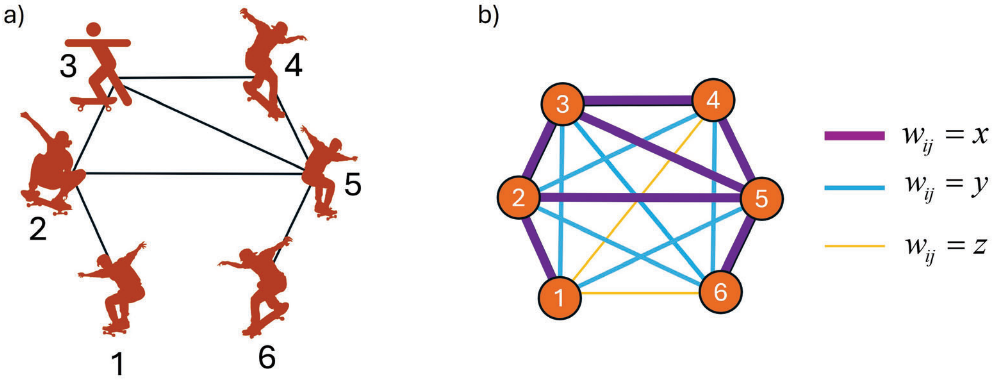

Fig. S1. (a) Illustration of a hypothetical network of direct influences between six skaters. (b) Graphical representation of the direct (edge of color magenta) and indirect (edges of blue and yellow color) influences between the six skaters forrming the network illustrated in a).

Mathematically we can represent the structure of this network by means of its adjacency matrix A:

$$A = \begin{pmatrix} 0 & 1 & 0 & 0 & 0 & 0 \\ 1 & 0 & 1 & 0 & 1 & 0 \\ 0 & 1 & 0 & 1 & 1 & 0 \\ 0 & 0 & 1 & 0 & 1 & 0 \\ 0 & 1 & 1 & 1 & 0 & 1 \\ 0 & 0 & 0 & 0 & 1 & 0 \end{pmatrix}.$$

This matrix indicates, for instance, that skater 1 can influence directly skater 2 as they are directly connected in the network. In a real-world situation this means that both skaters can discuss about their styles and share information about new trick which one of them has learned. If skater 2 adopts this new trick (an innovation in skateboarding) it can also discuss it with skater 3 and/or skater 5, who may be disposed to adopt it. This is the standard way in which “information” is supposed to be transmitted through the vertices of a network: the information is transmitted through the edges of the network.

If we assume, as frequently done, that two vertices connected in a network represent a kind of similarity or homophily, we should agree that skaters 1 and 2 have possibly more similar styles than the ones of skater 1 and 6. The reason is that skaters 1 and 6 are more “socially distant” in the network that skaters 1 and 2. Here we adopt the idea that the social distance can be captured by the topological (shortest path) distance between the vertices in the network. Although this is not necessarily always correct, it is a very good proxy, as if two people share a large set of similar values and attitudes they should be separated by only a few edges in their social networks, even if they are geographically distant.

Therefore, let us consider that social proximity can be represented by the shortest path distance in the network. In this way, A only represents the closest social proximity between pairs of individuals, i.e., those pairs of skaters that can discuss their styles and new tricks. However, on the similar basis we should agree that skaters 1 and 3 are more proximal among them than skaters 1 and 6. In terms of homophily we can think that the first pair of skater share more similarities in their styles and tricks used than the second pair. Therefore, if skater 1 has the chance of observing both skaters 3 and 6 on the skatepark it will be more prone to adopt some of the tricks of skater 3 than those of skater 6. At the end of the day, skater 1 seems more proximal to 3 than to 6. Then, if we represent all the pairs of skaters that are in a similar situation as skaters 1 and 3 we obtain the second social proximity matrix A_2. Similarly we can represent the third social proximity–which is the situation in which skaters 1 and 6 are–by A_3, and so forth:

$$A_2 = \begin{pmatrix} 0 & 0 & 1 & 0 & 1 & 0 \\ 0 & 0 & 0 & 1 & 0 & 1 \\ 1 & 0 & 0 & 0 & 0 & 1 \\ 0 & 1 & 0 & 0 & 0 & 1 \\ 1 & 0 & 0 & 0 & 0 & 0 \\ 0 & 1 & 1 & 1 & 0 & 0 \end{pmatrix}, A_3 = \begin{pmatrix} 0 & 0 & 0 & 1 & 0 & 1 \\ 0 & 0 & 0 & 0 & 0 & 0 \\ 0 & 0 & 0 & 0 & 0 & 0 \\ 1 & 0 & 0 & 0 & 0 & 0 \\ 0 & 0 & 0 & 0 & 0 & 0 \\ 1 & 0 & 0 & 0 & 0 & 0 \end{pmatrix}.$$

We have assumed that skater 1 can adopt a trick from skater 3 more easily than from skater 6, but we have not denied the possibility that it can adopt such a trick from the more distant skater. Therefore, our model assumes that one skater can adopt a new trick from any other member of the network, but with an influence that decay with their separation in the network. That is, if we consider the matrix of mutual influences as Wit will have the form: $W = xA + yA_2 + zA_3$ where $x > y > z$:

$$W = \begin{pmatrix} 0 & x & y & z & y & z \\ x & 0 & x & y & x & y \\ y & x & 0 & x & x & y \\ z & y & x & 0 & x & y \\ y & x & x & x & 0 & x \\ z & y & y & y & x & 0 \end{pmatrix}.$$

The classical network-theoretic approach just assumes that $x = 1$ and $y = z = 0$. Of course, W is the adjacency matrix of a weighted complete graph as illustrated in the Fig. S1(b). However, this complete graph is not any complete graph. It is one that "remembers" the original structure of the topological skeleton of the network. This is illustrated in Table S1 where we give the eigenvalues of W for the six nonisomorphic conneted graphs using $x = 1$, $y = 1/2$ and $z = 1/3$.

graph	λ_1	λ_2	λ_3	λ_4
a	2.2033	-0.0657	-0.8699	-1.2676
b	2.3028	-0.5	-0.5	-1.3028
c	2.5	-0.5	-0.5	-1.5
d	2.5358	-0.326	-1.0	-1.2098
e	2.7656	-0.5	-1.0	-1.2656
f	3	-1	-1	-1

Table S1. Eigenvalues of the matrix W as defined in this section for the six graphs in Fig. S2.

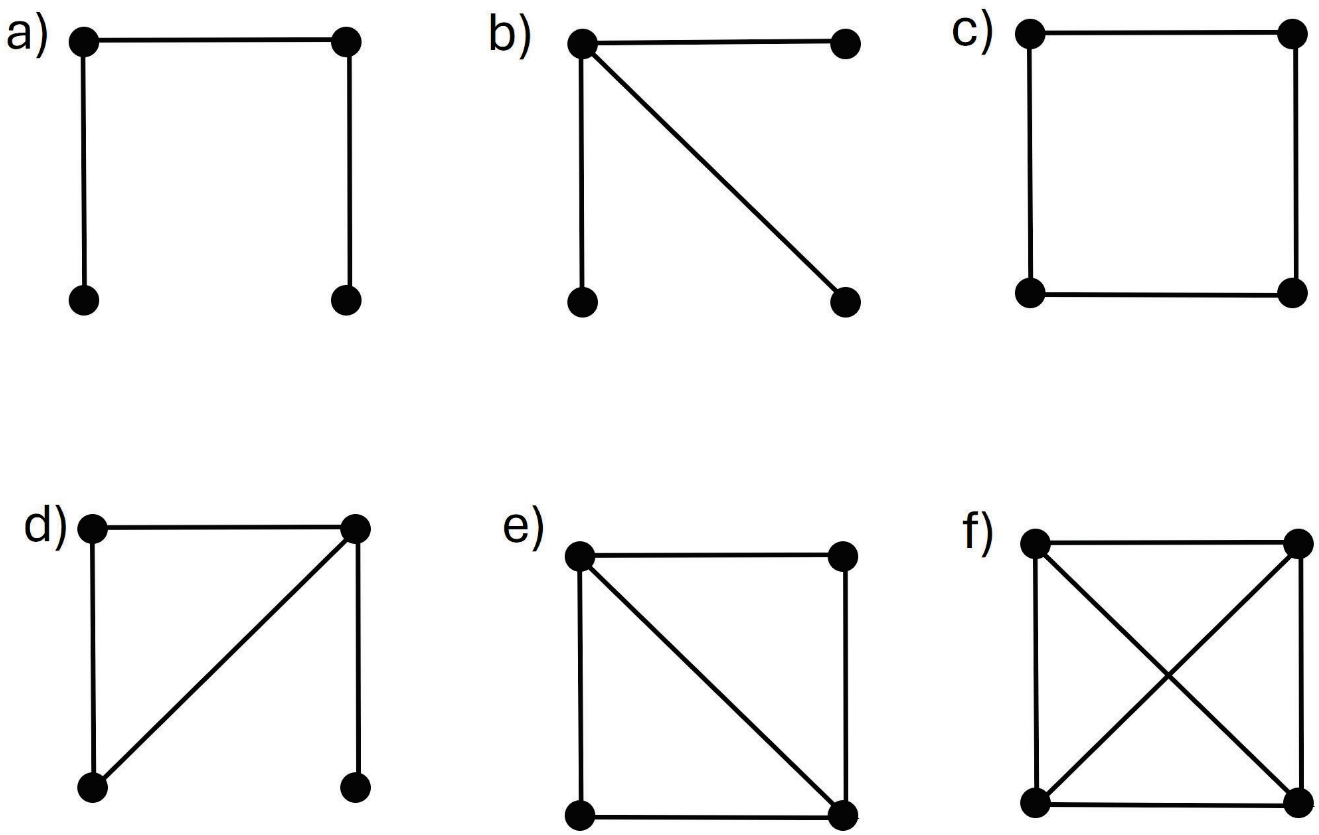

Fig. S2. The six nonisomorphic connected graphs with 4 vertices.

As stated in the main text of the paper the standard diffusion process on a network is modeled by the equation: $\dot{u}(t) = -\mathcal{L}_1 u(t)\,; u(0) = u^0$ where $\mathcal{L}_1 := K - A$, with K being the diagonal matrix of vertex degrees. The model accounting for both direct and indirect influences (Eq. (4) in the main text) can be written with the same initial condition as before as:

$$\dot{u}(t) = -\left(\mathcal{L}_1 + \sum_{d=2}^{D} c_d \mathcal{L}_d\right) u(t) \quad [1]$$

where $\mathcal{L}_d \coloneqq K_d - A_d$ in which A_d has been defined before and $K_d \coloneqq \operatorname{diag}\left(A_d \vec{\mathbf{1}}\right)$ ($\vec{\mathbf{1}}$ is a column vector of ones). By using the matrix of influences W defined before we can write:

$$\dot{u}(t) = -\mathcal{L}u(t), \quad [2]$$

such that $\mathcal{L} \coloneqq \left(\operatorname{diag}\left(W\vec{\mathbf{1}}\right) - W\right)$:

$$\mathcal{L} = \begin{pmatrix} x+2y+2z & -x & -y & -z & -y & -z \\ -x & 3x+2y & -x & -y & -x & -y \\ -y & -x & 3x+2y & -x & -x & -y \\ -z & -y & -x & 2x+2y+z & -x & -y \\ -y & -x & -x & -x & 4x+y & -x \\ -z & -y & -y & -y & -x & x+3y+z \end{pmatrix}.$$

Obviously when $x = 1$ and $y = z = 0$ then $\mathcal{L} = \mathcal{L}_1$.

To illustrate how $\mathcal{L}$ captures significantly more structural information that $\mathcal{L}_1$ and how this influences a diffusion process we consider the two graphs illustrated in Fig. S3. These two graphs are Laplacian cospectral, which mean that they have identical spectra for the Laplacian matrix. Indeed, the eigenvalues of $\mathcal{L}_1$ for both graphs are: $0, 0.7639, 2, 3, 3, 5.2361$.

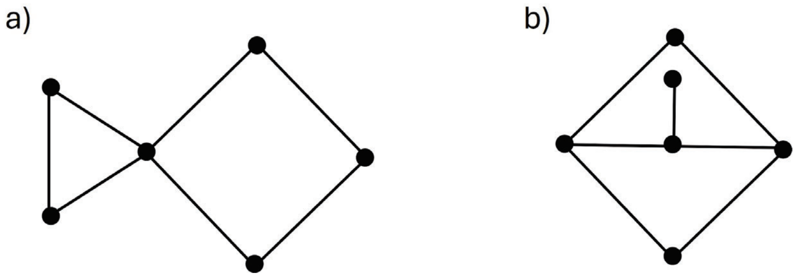

Fig. S3. Illustration of a pair of graphs which are Laplacian cospectral.

It is well-known that if we desigate by $0{=}\mu_1 < \mu_2 \leq \cdots \leq \mu_n$ be the eigenvalues of $\mathcal{L}_1$ and by φ_i the orthonormalized eigenvector associated with μ_i, we can write

$$\begin{aligned} u(t) &= e^{-t\mathcal{L}_1} u^0 \\ &= \varphi_1 e^{-t\mu_1}\left(\varphi_1^T u^0\right) + \varphi_2 e^{-t\mu_2}\left(\varphi_2^T u^0\right) + \cdots + \varphi_n e^{-t\mu_n}\left(\varphi_n^T u^0\right). \end{aligned} \quad [3]$$

When t is sufficiently large we have

$$\begin{aligned} \lim_{t\to\infty} u(t) &= \varphi_1 e^{-t\mu_1}\left(\varphi_1^T u^0\right) \\ &= \frac{1}{\sqrt{n}}\vec{\mathbf{1}}\left(\frac{1}{\sqrt{n}}\vec{\mathbf{1}}^T u^0\right) \\ &= \vec{\mathbf{1}}\left(\frac{1}{n}\sum_{i=1}^{n} u_i^0\right). \end{aligned} \quad [4]$$

Because μ_2 is the second smallest eigenvalue of $\mathcal{L}_1$, it will dictate the rate of convergence of the process. This means that the rate of convergence of a standard diffusion process on the two graphs on Fig. S3 is exactly the same.

However, if we consider the diffusion under the combined effects of direct plus indirect influences we will have

$$\begin{aligned} u(t) &= e^{-t\mathcal{L}} u^0 \\ &= \phi_1 e^{-t\sigma_1}\left(\phi_1^T u^0\right) + \phi_2 e^{-t\sigma_2}\left(\phi_2^T u^0\right) + \cdots + \phi_n e^{-t\sigma_n}\left(\phi_n^T u^0\right), \end{aligned} \quad [5]$$

where $0{=}\sigma_1 < \sigma_2 \leq \cdots \leq \sigma_n$ are the eigenvalues of $\mathcal{L}$ and ϕ_j the corresponding orthonormalized eigenventors. Then, when t is sufficiently large we have

$$\lim_{t\to\infty} u(t) = \phi_1 e^{-t\sigma_1}\left(\phi_1^T u^0\right)$$
$$= \frac{1}{\sqrt{n}}\vec{\mathbf{1}}\left(\frac{1}{\sqrt{n}}\vec{\mathbf{1}}^T u^0\right) \qquad [6]$$
$$= \vec{\mathbf{1}}\left(\frac{1}{n}\sum_{i=1}^{n} u_i^0\right),$$

and because σ_2 is the second smallest eigenvalue of $\mathcal{L}$, it will dictate the rate of convergence of the process. The final state of the process is exactly the same as for the case with direct influences only. However, the eigenvalues of $\mathcal{L}$ for graph (a) in Fig. S3 are: $0, 3, 4, 4.333, 4.4226, 5.5774$ and those for graph (b) are $0, 2.9350, 3.8333, 4.50, 4.50, 5.5650$. Therefore, the two graphs are not isospectral for $\mathcal{L}$ and more importantly, graph a) has a larger value of σ_2 than graph b), which means that a diffusion process under the direct plus indirect influences will converge in shorter time in the graph a) than in b). Here it is an example where the consensus among the agents with direct influences only differ significantly in both qualitative and quantitative terms from the one using combined direct+indirect influences. The nontriviality of the result can be seen by the fact that both graphs have the same number of vertices (6), the same number of edges (7), the same diameter (3), the same number of pairs of vertices at distance 2 (6) and the same number of pairs of vertices at distance 3 (2).

2. Experiment description

Our experiment was divided into 5 sequential phases: Instructions, the first setting (only short-range interactions) and three differently ordered settings (mixing short and long-range interactions). A schematic workflow of the procedure for each Settings is shown in Fig. S4.

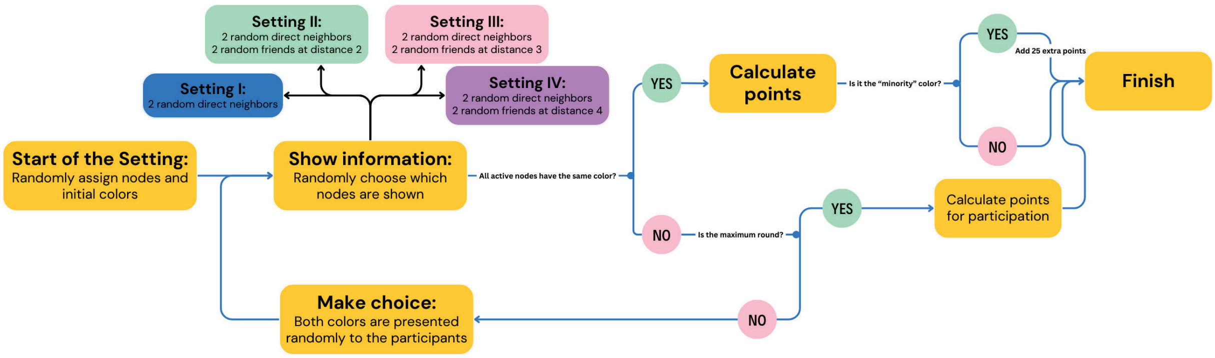

Fig. S4. Workflow for implementing each Setting in the experiment.

In the Instructions (Fig. S5), we explained the participants the motivation of the experiment and gave them the rules, objective and payment during the session. We explained them that our objective was to study how an innovation spreads through a social network, based on the situation studied in (1).In this study, known as the Columbia University Drug Study, researchers collected data on the first subscription to the drug "gammanym" by 31 physicians in several communities. The doctors are related via a face-to-face social network of friendship and discussion, which was created by asking them to name three doctors whom they considered to be personal friends and to nominate three doctors with whom they would choose to discuss medical matters. We explained that, along the experiment, each of the participants would represent one of the doctors from the original study, linked with the same social relations. We made minor modifications to the graph to make sure that every participant has at least two nearest neighbors and that all of them have at least two other participants at distance four. It was needed only to rewire one edge, thus the network has exactly the same density as the original one and about the same topology.

The participants have to choose each round between two different colors, representing two different drugs to prescribe, and one of them will be an innovation which will be a minority at the beginning. To avoid any uncontrollable bias towards the word "innovation", we referred to such color as the minority color at the beginning. The objective of the participants is for all of them to choose the same color as soon as possible. The participant were told that each setting would consist in a number of rounds between 10 and 15, although we always chose random numbers between 13 and 15. Each participant would win 1 point per round in which they took a decision and 5 points for each round left between the consensus round and the maximum round, plus 15 points if the consensus was achieved in the innovation color (minority at the beginning).

After these explanations, we showed them an image similar to the networks where we will show them the information (Fig. S6). They are networks displayed in circular layers, such that the central node corresponds to the participant and the rest of nodes are disposed in further layers depending on the topological distance to the central one. To visually distinguish easily the distance of each layer, we plotted gray figures in the background which were darker as they were closer to the central node.

Furthermore, a text indicating which node is the player is displayed on top of the central node. For each node in the network, two types of information are shown. Firstly, the color chosen by such node, being black if that information is not provided to the player. Secondly, the shape of the node is a circle if it represents an active player (who has made a decision in this round) or a triangle if the represented player is inactive.

To capture if instructions are clearly understood, 4 questions had to be answered by the participants: Which color have you chose this round?, Which color was chosen by your friends?, Which color was chosen by the friends or your friends, excluding yourself? and How many inactive players are there in this round?. The first three questions had options "Blue", "Yellow" and "We can't know it", while the last one had to be answered by typing a number. After answering them, the next page showed if they answered correctly each question and, if they were wrong, they were given a brief explanation about which was the correct answer and why (Fig. S7).

After all players completed the Instructions phase, the Setting I started. The first pages was a brief explanation of this game (Fig. S8a), indicating that in this phase there will be several rounds and in each of them we will show them two of their friends (nodes at distance 1). This friends are chosen randomly, excluding inactive players if possible. They were also told between which two colors they had to choose (Blue and Yellow), which of them was the initially minority color for which we would pay them more points (Blue) and which color was initially assigned to them. After that, successive pages of the resulting network (Fig. S8b) and a choice page (Fig. S8c), in which the two colors were displayed in random order to each participant in each round. If the choice of all participants is the same or if the maximum number of rounds is achieved, a final page with the result and points earned is shown (Fig. S8d).

Then, the three next phases consisted in Settings II, III and IV, differently ordered for each of the experiment sessions. In each of them, the same sequence of pages are shown, as for Setting I (an explanation, several rounds of the resulting network and the choice page and finally a result page with the points earned). In Setting II there were shown two random neighbors (at distance 1) and two random nodes at distance 2, always evading inactive players if possible. In this setting the colors to chose between are Green and Magenta, being the last one the minority color at the beginning. In Setting III there were shown also two random neighbors at distance 1 and two random nodes at distance 3, choosing between Orange (minority) and Red (majority). Finally, in Setting IV participants had to choose between Purple as minority color and Lilac, and we showed them two nodes at distance 1 and two nodes at distance 4. Explanation pages are shown in Figs. S9a,S10a,S11a and examples of the Network pages are in S9b,S10b,S11b.

Finally, the last pages shown to the participants (Figs. S12a,S12b) gave each participant the total sum of points they earned, its equivalence in euros and asked them to introduce their Paypal account. After that, a greeting final page was shown.

Bienvenido al experimento

Tiempo disponible para completar esta página: **2:07**

Instrucciones

Este experimento pretende recrear la elección de diferentes medicamentos por los médicos de un hospital. Cada uno de los participantes representará a un médico, que mostraremos como un círculo, y los dos medicamentos entre los que tiene que elegir serán representados por colores dentro de los círculos durante este experimento. Al igual que los médicos tenían distintos amigos médicos dentro del hospital, en cada uno de los bloques de este experimento usted estará conectado con los participantes que representan a los médicos amigos, y podrá ir viendo qué colores han elegido.

Tomará varias decisiones de forma consecutiva, en momentos de tiempo que llamaremos “rondas” del experimento. Para mostrar los resultados después de cada ronda, verá una imagen interactiva. En la imagen, usted aparecerá como un círculo en el centro, del color que haya elegido. A una distancia más cercana a usted, en un primer círculo gris, aparecen sus amigos, seguido de un círculo más claro con los amigos de sus amigos, y así en adelante. Los puntos conectados por líneas son amigos directos. Podrá situar el cursor sobre cada punto para que se iluminen sus amigos, ya que en la representación puede haber líneas superpuestas.

Si algún participante no toma la decisión en el tiempo asignado, su representación ya no será un punto sino que tendrá forma triangular, indicando que su decisión se ha tomado de manera automatizada esa ronda. Estos participantes inactivos tienen un 50% de probabilidad de escoger un color aleatoriamente y otro 50% de probabilidad de escoger el color que más le acerque al consenso.

Cada bloque constará de entre 10 y 15 rondas. En cada una de estas rondas tendrá que escoger uno de los colores, viendo los resultados justo después. En cada ronda le mostraremos información de distintos puntos. El resto de nodos se mostrarán en negro y no tendrá información sobre ellos. Es decir, los nodos negros también han escogido un color cada ronda, pero usted no sabrá cuál es.

Su objetivo será conseguir que todos los participantes elijan el mismo color. Cuando lleguen al consenso, recibirá 5 puntos por cada ronda que quede para llegar al final. Recibirá 1 punto por cada ronda en la que tome usted la decisión. Además, se pagará un plus de 15 puntos si el color en el que se llega al consenso era el minoritario al comienzo.

Si no pulsa en el botón "Siguiente" antes de que pase el tiempo establecido en la página de la elección, esa ronda usted contará como inactivo. Para que le paguemos en cada ronda debe haber tomado una decisión y no estar inactivo.

Como ejemplo, si todos los participantes se ponen de acuerdo en la ronda 6 de 10, cada uno ganará 6 puntos por las rondas participadas más 5x4=20 puntos por haber llegado al consenso 4 rondas antes del final, sumando un total de 26 puntos. Si además el color al llegar al consenso era el minoritario, cada participante recibirá 15 puntos extra, llegando a tener 41 puntos en total.

Ahora responderá algunas preguntas de control que le ayudarán a comprender las instrucciones. Podrá volver a leer un resumen de las instrucciones en las páginas que siguen. Pulse "Siguiente" cuando esté listo para continuar.

Siguiente

IBSEN Bridging the gap: from Individual Behavior to the Socio-Technical Man

HORIZON 2020

Fig. S5. Instructions given at the start of the experiment.

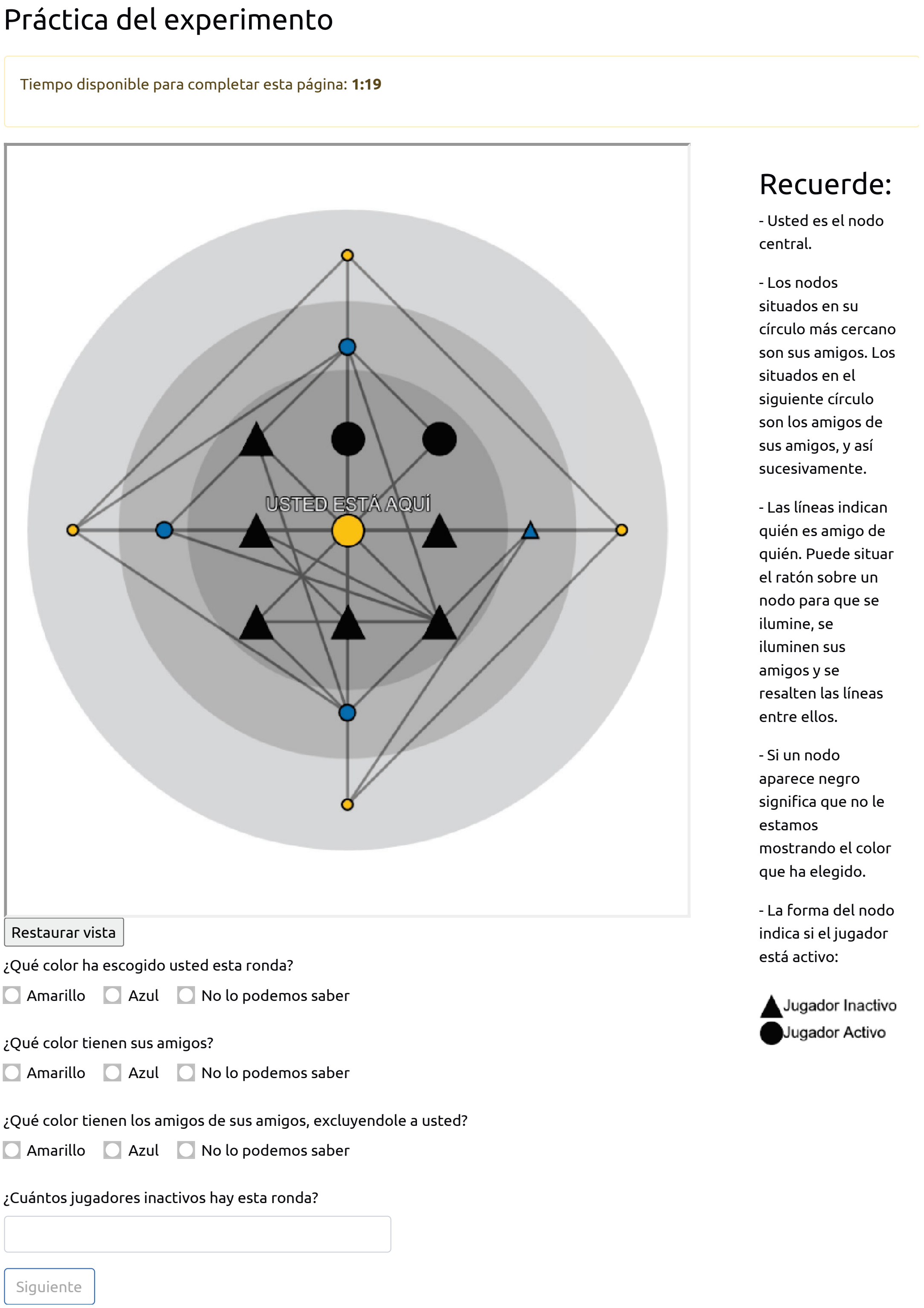

Fig. S6. Practice network shown in the Instructions phase.

Práctica del experimento

Tiempo disponible para completar esta página: **1:57**

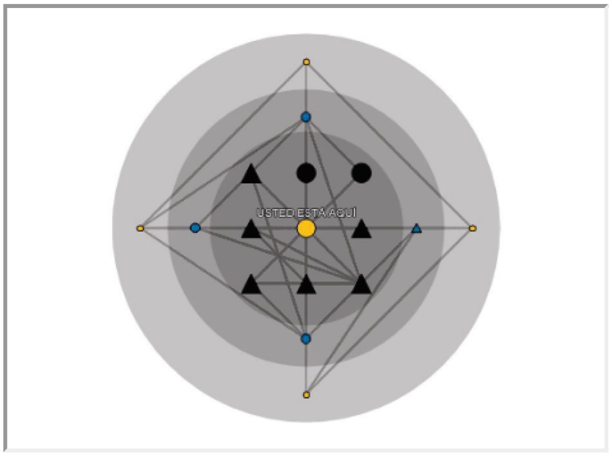

- ¿Qué color ha escogido usted esta ronda?

- Falló en la primera pregunta. Usted es el nodo que se encuentra en el centro, bajo las letras "USTED ESTÁ AQUÍ". Ese nodo tiene el color Amarillo en esta imagen.

- ¿Qué color tienen sus amigos?

- Falló en la segunda pregunta. Sus amigos son los que se encuentran en el círculo más cercano a usted. Si un nodo tiene color negro significa que no le estamos mostrando su decisión, por lo que no se puede saber qué color ha elegido ese participante.

- ¿Qué color tienen los amigos de sus amigos?

- Falló en la tercera pregunta. Los amigos de sus amigos son los que se encuentran en el segundo círculo. En esta imagen todos ellos tienen el color Azul.

- ¿Cuántos jugadores inactivos hay esta ronda?

- Falló en la cuarta pregunta. Los jugadores inactivos son aquellos cuyos nodos tienen forma de triángulo en vez de círculo. En esta imagen hay 7 triángulos.

Siguiente

Fig. S7. Explanation of the wrong answers to the questions in the Instructions phase.

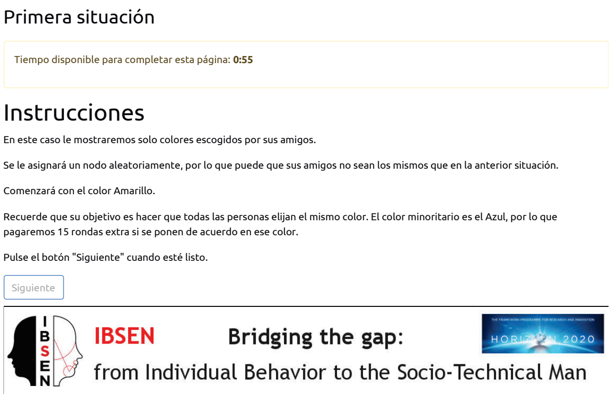

(a) Explanation of the game for Setting I.

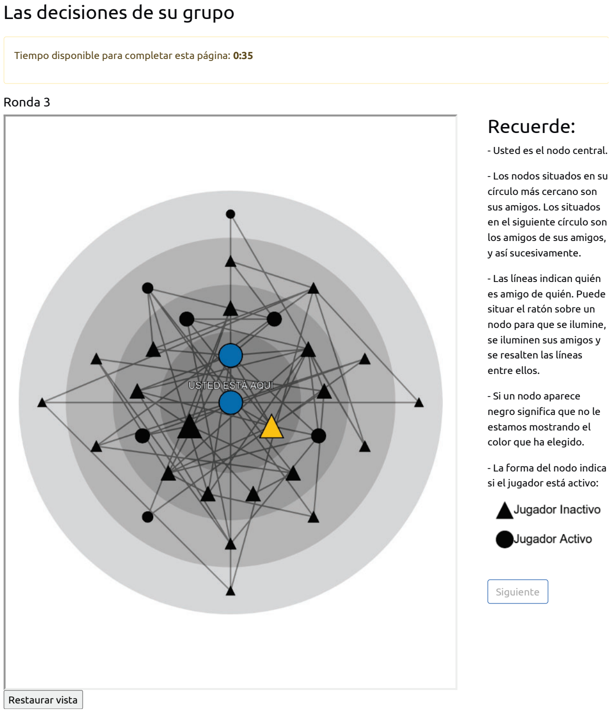

(b) Resulting network page for Setting I.

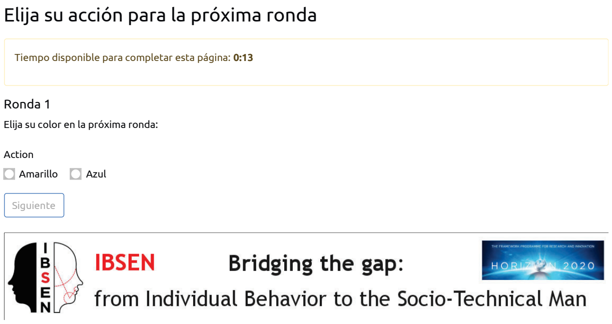

(c) Choice page for Setting I.

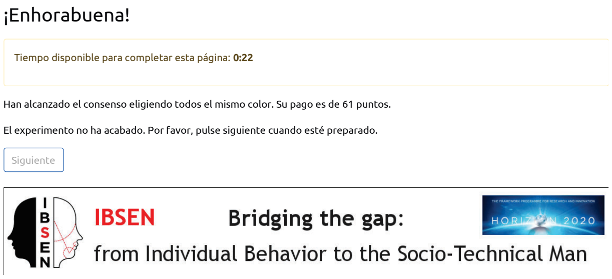

(d) Results page for Setting I.

Fig. S8. Overview of the different pages for Setting I.

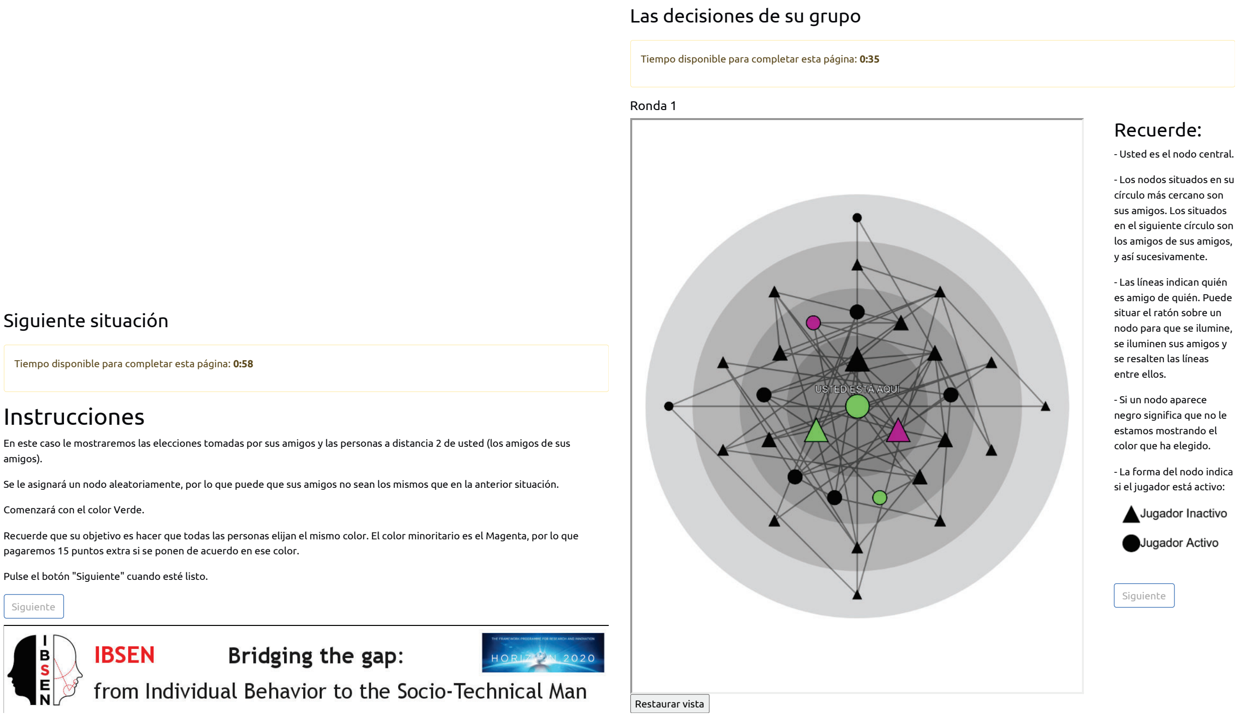

(a) Explanation of the game for Setting II. **(b)** Resulting network shown for Setting II.

Fig. S9. Overview of explanation and network for Setting II.

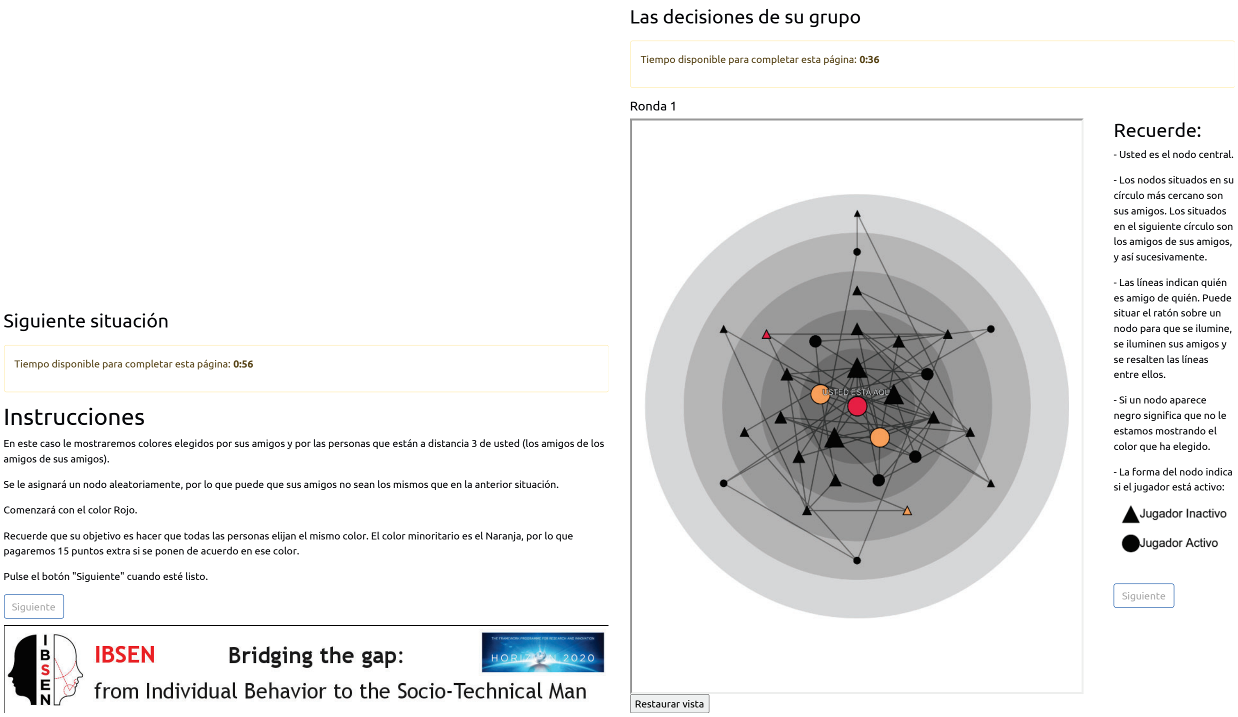

(a) Explanation of the game for Setting III. **(b)** Resulting network shown for Setting III.

Fig. S10. Overview of explanation and network for Setting III.

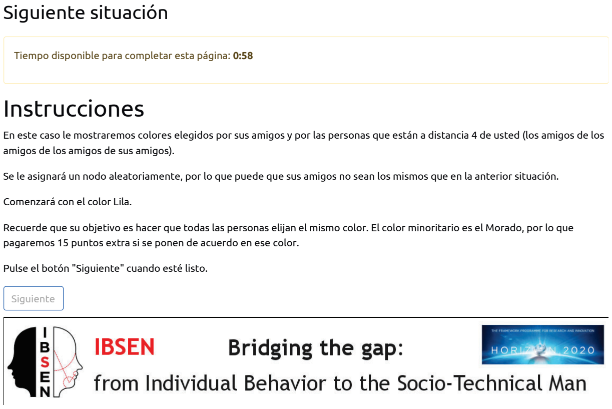

(a) Explanation of the game for Setting IV.

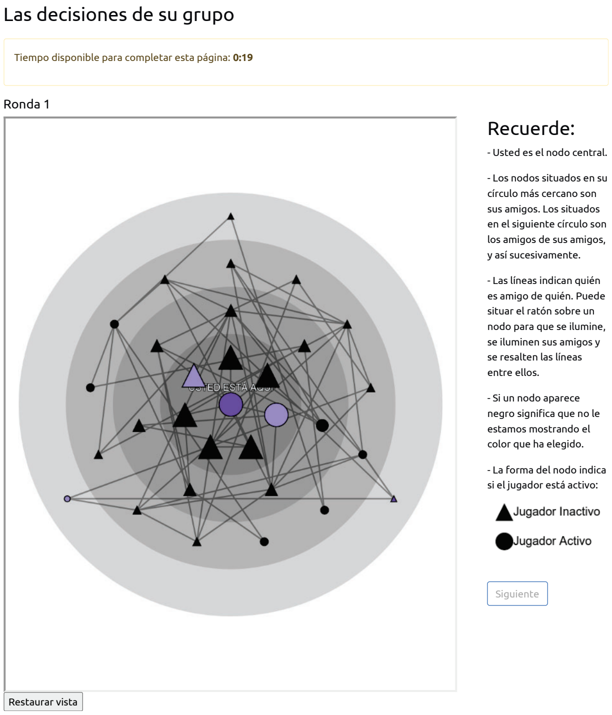

(b) Resulting network shown for Setting IV.

Fig. S11. Overview of explanation and network for Setting IV.

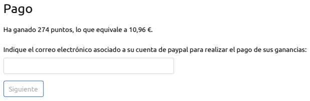

(a) Payment page.

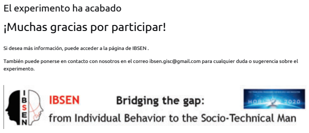

(b) End-page.

Fig. S12. Payment and end-page.

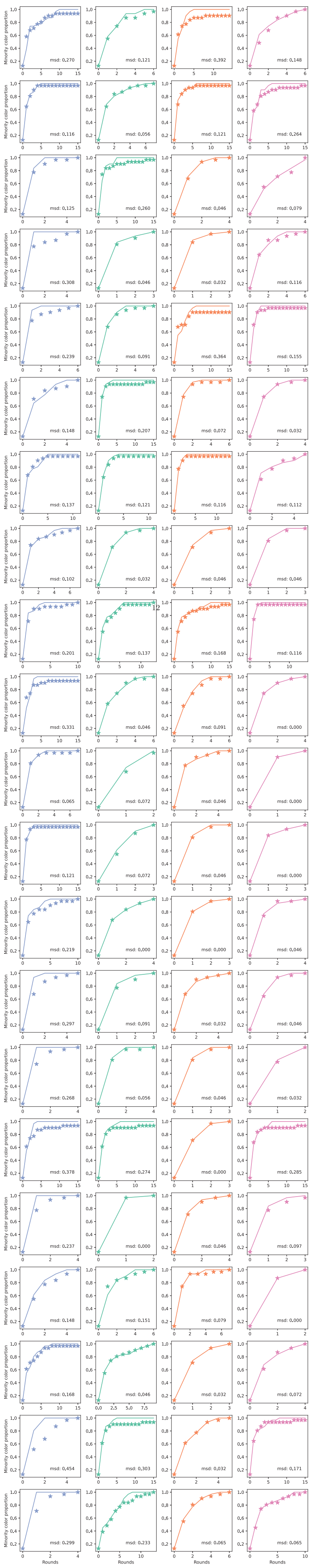

Fig. S13. Individual results and fitted curves for each session and Setting of the experiment.

3. Model adjustment with the median

In the Experimental Results section, we assumed that, although our data does not look normally distributed, it was due to the small size effect, and the real distribution would be Normal ones. Nonetheless, we could perform a different approach without considering this normality assumption. For that purpose, instead of the mean and standard deviation, we should use the medians ($c_2 = 0.68$, $c_3 = 0.1$, $c_4 = 0.64$). As we did in the article with the t-test, we can compare the distributions using the Mann-Whitney U test. The p-values obtained are: p-value$(c_2, c_3) = 0.0524$, p-value$(c_2, c_4) = 0.1694$ and p-value$(c_3, c_4) = 0.87$. As well as it occurs with the normality assumptions and the t-test, we cannot statistically ensure that medians are not equal pairwise, but the p-value for the distributions of c_2 and c_3 is close to the standard limit value of 0.05, so it may be considered valid due to the small sample size.

Thus, we discard Setting IV as the less conclusive one from our data and use Settings II and III to do our median prediction model: $\dot{u}(t) \approx -(\mathcal{L}_1 + 0.68\mathcal{L}_2 + 0.1\mathcal{L}_3)u(t)$. In Fig.S14 we plot the mean behavior of the classical model (using only $\mathcal{L}_1$), our model with the mean parameters used in the main text and our model with the median values presented here, randomizing the initial conditions. We can observe that the change between these two long-range models is minimum when picking the mean or the median values.

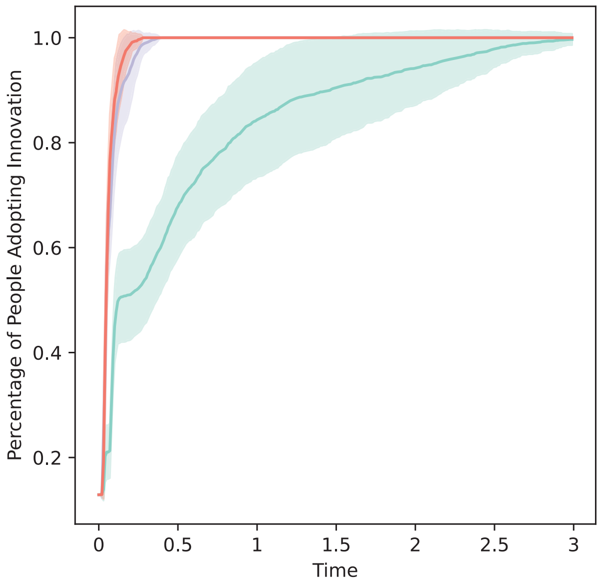

Fig. S14. Mean plot of different diffusion models using 1000 random initial conditions. Green line represents the classical (short distance) model, gray line represents the long-range model with the median parameters and red line represents the same model but with the mean parameters.

4. Other possible coefficient decays for long-range interactions

For the d-path Laplacian operators, there has been two types of transformations that have been studied (2, 3): Mellin transformation, that corresponds to coefficients $c_d = d^{-s}$ for $s > 0$, and Laplace transformation, whose coefficients are given by $c_d = e^{-d\lambda}$ for $\lambda > 0$. The first one has been more deeply investigated as it was proven that, for certain values of the parameter s, it models a superdiffusive process in a 1-dimensional infinite path graph or in a 2-dimensional infinite square lattice.

We calculated which values of the parameters s and λ would correspond to each of the experiments and Settings within them (Figs. S15 and S16).

Focusing on the s parameters, we obtain that, in both cases, the median of Setting II and IV are similar ($s = 0.21$ and $s = 0.27$, respectively), while for Setting III it increases to $s = 2.08$. This reflects the fact that in Setting III there could be spotted two different groups of values for c_3, one with values close to 1 and other with lower values, between 0 and 1/3. It is the first of them that pushes the median value higher, which means a curve slowly decaying (further nodes are still considered as important).

Another relevant note when considering such functions is to not group by Settings for fitting. This is, if we calculated the mean obtained parameter for each of the sessions, the best parameter to fit that data would be the mean of the values obtained. For our experiments, this would give a value $s = 1.63 > 1$. On the other hand, we could fit the function parameter to the brute data c_d, which in our case gives $s = 0.65 < 1$. This difference (see Fig. S17), result of the logarithmic transformations needed to go from c_d to the s parameters, shows that, depending on the contexts (Settings), the same group of people may consider peer influence with different rules (decay functions). This should be considered in future researches replicating or expanding these experiments.

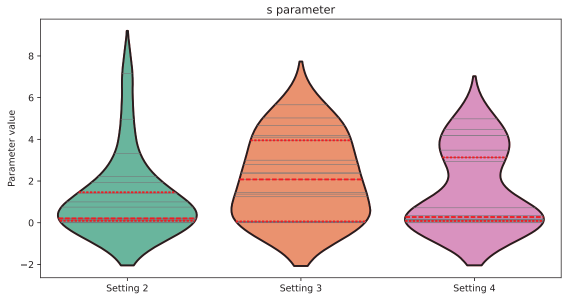

Fig. S15. Distribution of the values of the s parameters for each of the sessions and Settings.

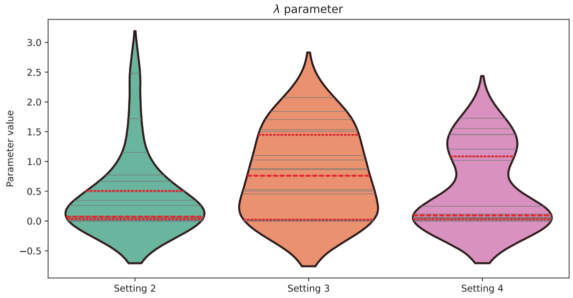

Fig. S16. Distribution of the values of the λ parameters for each of the sessions and Settings.

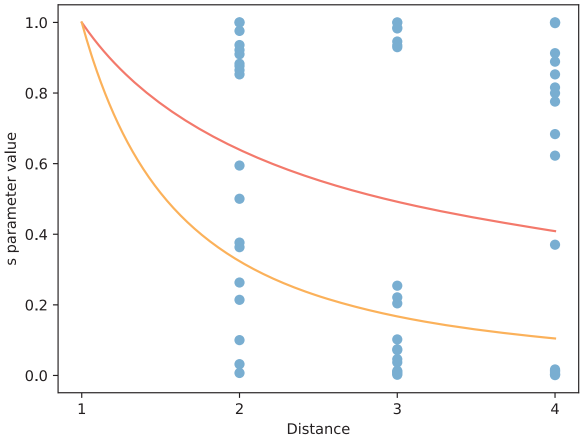

Fig. S17. Power-law fitting of the data (blue dots) by MSE (red line) and by the mean of the transformed s parameters (orange line).

5. Demographics

We recruited 596 participants in 21 sessions completed along 4 months, plus 3 testing sessions and 2 more canceled ones. The number of active participants in total was 596, who received a mean payment of 4, 50€. 63.8% of the were female, 35. 9% were male and 0. 3% were non-binary. The average age was 30.40 (sd = 11.22). The age distribution of the participants per gender is shown in the following table and figures.

Table S2. Gender distribution per experimental session

Gender	Total	Experimental session																				
		1	2	3	4	5	6	7	8	9	10	11	12	13	14	15	16	17	18	19	20	21
Female	380	14	18	17	17	19	15	19	21	22	22	21	12	19	18	19	17	19	18	16	16	21
Male	214	12	8	13	11	9	13	8	6	9	8	10	9	12	11	12	13	11	8	14	10	7
Non-binary	2										1								1			

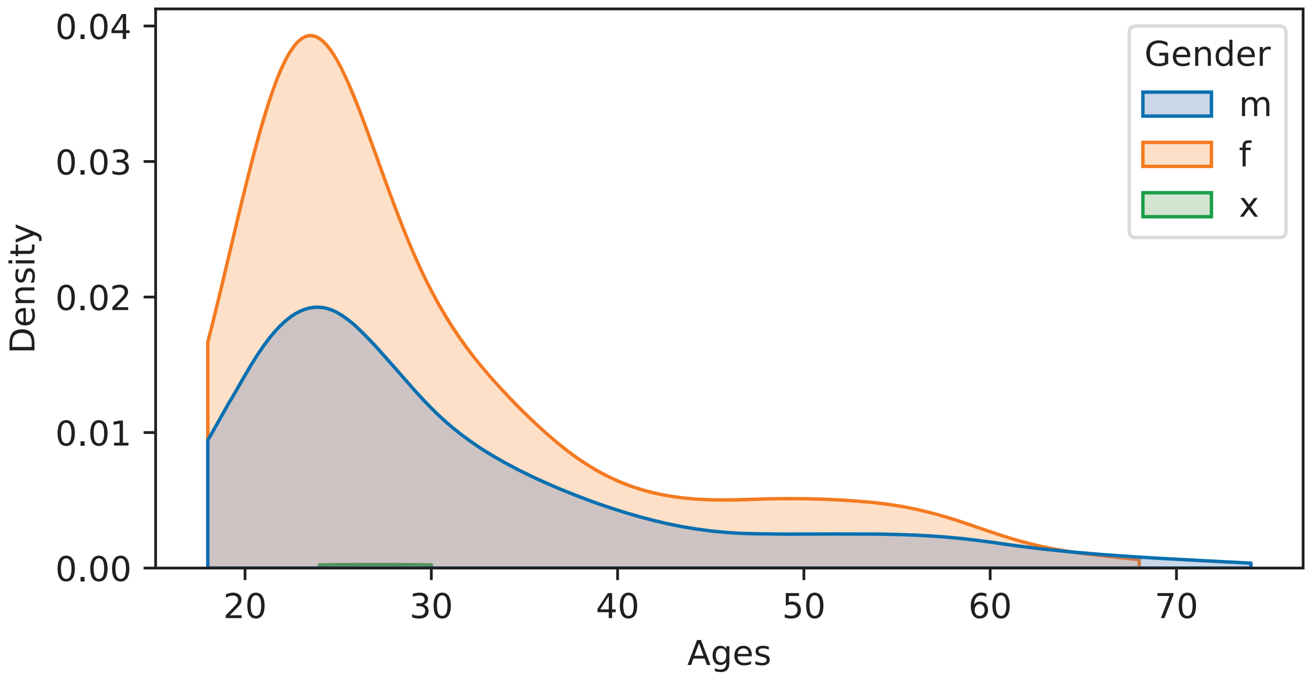

Fig. S18. Probability density function (PDF) of participant ages, per gender (m: male, f: female, x: non-binary. PDFs per gender are scaled by the number of observations such that the total area under all densities sums up to 1.

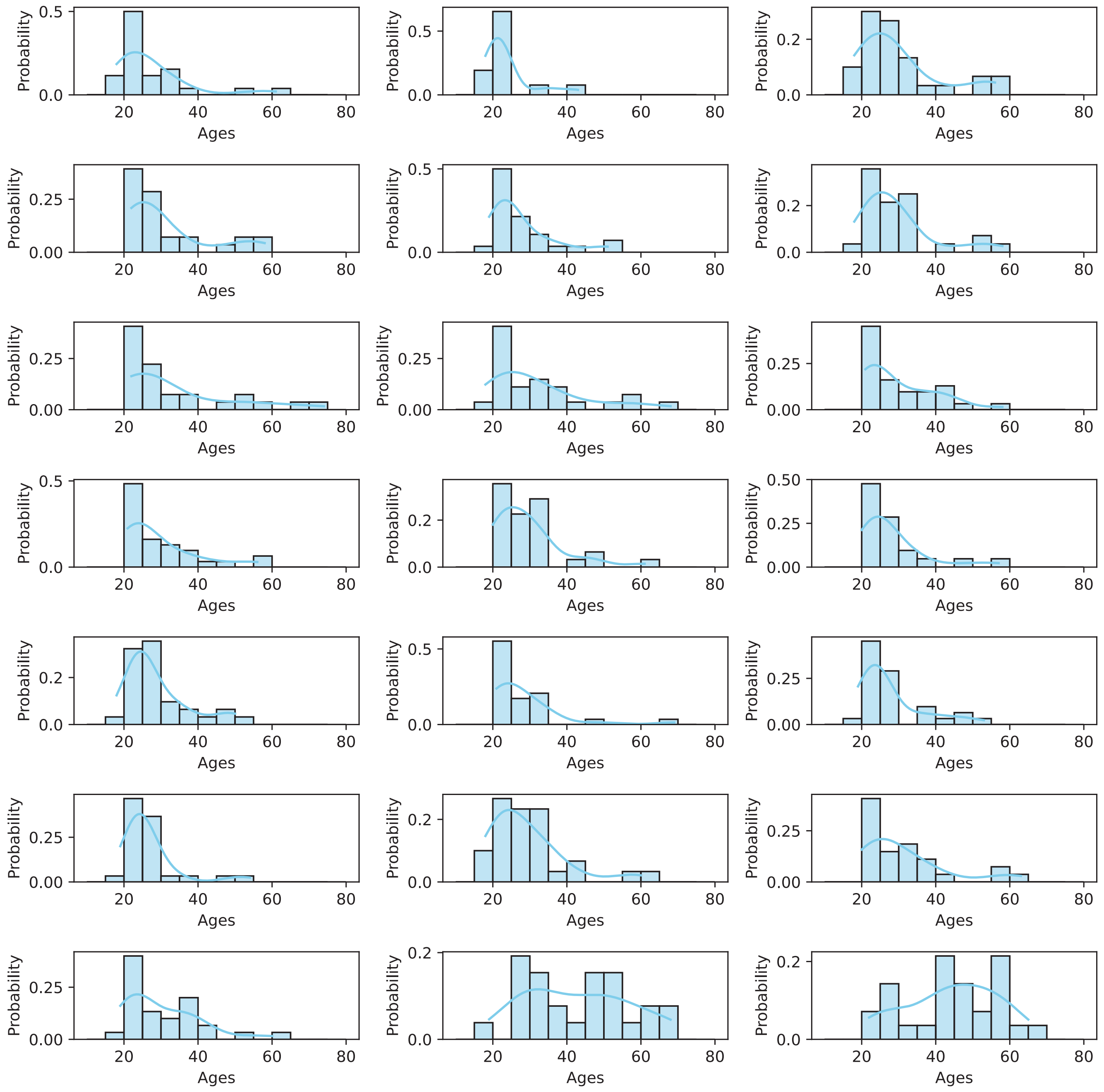

Fig. S19. Age probability function per experimental session. From top-left (session 1) to bottom-right (session 21). Bins width is five years.

References

1. H Menzel, E Katz, Social relations and innovation in the medical profession: The epidemiology of a new drug. *Public opinion quarterly* **19**, 337 (1955).
2. E Estrada, EM Hameed, N Hatano, M Langer, Path laplacian operators and superdiffusive processes on graphs. i. one-dimensional case. *Linear algebra its applications* **523**, 307–334 (2017).
3. E Estrada, EM Hameed, M Langer, A Puchalska, Path laplacian operators and superdiffusive processes on graphs. ii. two-dimensional lattice. *Linear algebra its applications* **555**, 373–397 (2018).
